# Prophylactic antibiotics in the prevention of infection after operative vaginal delivery (ANODE): a multicentre randomised controlled trial

**DOI:** 10.1016/S0140-6736(19)30773-1

**Published:** 2019-06-15

**Authors:** Marian Knight, Virginia Chiocchia, Christopher Partlett, Oliver Rivero-Arias, Xinyang Hua, Kim Hinshaw, Derek Tuffnell, Louise Linsell, Edmund Juszczak, ANODE collaborative group, Marian Knight, Helen Enderby, Derek Tuffnell, Kim Hinshaw, Ranee Thakar, Abdul H Sultan, Julia Sanders, Dharmintra Pasupathy, Philip Moore, James Gray, Oliver Rivero-Arias, Edmund Juszczak, Louise Linsell, Aethele Khunda

**Affiliations:** a National Perinatal Epidemiology Unit, Nuffield Department of Population Health, University of Oxford, Oxford, UK; b City Hospitals Sunderland NHS Foundation Trust and Faculty of Health Sciences, University of Sunderland, Sunderland, UK; c Bradford Teaching Hospitals NHS Foundation Trust, Bradford, UK

## Abstract

**Background:**

Risk factors for maternal infection are clearly recognised, including caesarean section and operative vaginal birth. Antibiotic prophylaxis at caesarean section is widely recommended because there is clear systematic review evidence that it reduces incidence of maternal infection. Current WHO guidelines do not recommend routine antibiotic prophylaxis for women undergoing operative vaginal birth because of insufficient evidence of effectiveness. We aimed to investigate whether antibiotic prophylaxis prevented maternal infection after operative vaginal birth.

**Methods:**

In a blinded, randomised controlled trial done at 27 UK obstetric units, women (aged ≥16 years) were allocated to receive a single dose of intravenous amoxicillin and clavulanic acid or placebo (saline) following operative vaginal birth at 36 weeks gestation or later. The primary outcome was confirmed or suspected maternal infection within 6 weeks of delivery defined by a new prescription of antibiotics for specific indications, confirmed systemic infection on culture, or endometritis. We did an intention-to-treat analysis. This trial is registered with ISRCTN, number 11166984, and is closed to accrual.

**Findings:**

Between March 13, 2016, and June 13, 2018, 3427 women were randomly assigned to treatment: 1719 to amoxicillin and clavulanic acid, and 1708 to placebo. Seven women withdrew, leaving 1715 in the amoxicillin and clavulanic acid group and 1705 in the placebo groups. Primary outcome data were missing for 195 (6%) women. Significantly fewer women allocated to amoxicillin and clavulanic acid had a confirmed or suspected infection (180 [11%] of 1619) than women allocated to placebo (306 [19%] of 1606; risk ratio 0·58, 95% CI 0·49–0·69; p<0·0001). One woman in the placebo group reported a skin rash and two women in the amoxicillin and clavulanic acid reported other allergic reactions, one of which was reported as a serious adverse event. Two other serious adverse events were reported, neither was considered causally related to the treatment.

**Interpretation:**

This trial shows benefit of a single dose of prophylactic antibiotic after operative vaginal birth and guidance from WHO and other national organisations should be changed to reflect this.

**Funding:**

NIHR Health Technology Assessment programme.

## Introduction

Sepsis causes 11% of maternal deaths globally;^1^ in 2016, an estimated 19 500 women died because of pregnancy-related infections.^2^ This burden is not limited to countries of low and middle income; 5% of maternal deaths in high-income settings are due to infection, and in the USA this figure has been estimated to be as high as 13%.^3^ For every woman that dies from pregnancy-related infection, 70 women have severe infection and survive, often with long-term health consequences.^4^ Maternal infection remains under-recognised and undertreated, and hence is the focus of a WHO global study and awareness campaign.^5^

Risk factors for maternal sepsis can be easily recognised, including caesarean section and operative vaginal birth (forceps or vacuum extraction).^4^, ^6^ Without prophylaxis, an estimated 20–25% of women have an infection following caesarean birth,^7^ and up to 16% have an infection following operative vaginal birth.^8^ There is strong evidence from a systematic review^7^ in women giving birth by caesarean section, that the use of prophylactic antibiotics reduces the incidence of wound infection, endometritis, and serious maternal infection by 60–70%. Antibiotic prophylaxis at caesarean section is therefore widely recommended.^9^ Another systematic review^10^ identified only one small, low-quality evidence trial of antibiotic prophylaxis in operative vaginal birth, reporting only two outcomes, endometritis and maternal length of hospital stay, with no difference between women receiving an antibiotic and those receiving placebo.^11^ Recognising the importance of antibiotic stewardship where evidence of benefit is insufficient, current guidelines explicitly state that routine antibiotic prophylaxis is not recommended for women undergoing operative vaginal birth.^9^, ^12^, ^13^, ^14^

Although numbers of caesarean births are increasing worldwide,^15^ and in many countries the proportion of women undergoing operative vaginal birth has decreased,^16^ operative vaginal birth is recognised to be able to be accomplished safely and more quickly than caesarean birth, and avoids the associated complications.^16^ Recognition of the need to reduce unnecessary caesarean birth is increasing,^17^ which makes the impetus to effectively reduce morbidity after other modes of operative birth greater. Given the insufficient evidence of benefit of antibiotic prophylaxis after operative vaginal birth, we therefore did a randomised controlled trial to investigate whether a single dose of prophylactic amoxicillin and clavulanic acid was clinically and cost-effective for preventing confirmed or suspected maternal infection and other maternal outcomes.

**Research in context**

**Evidence before this study**

We searched PubMed on Dec 7, 2018, for all studies about operative vaginal birth and antibiotic prophylaxis, using the search terms “forceps”, “ventouse”, “vacuum extraction”, “antibiotic”, “prophylaxis”, “infection”, and “sepsis”, published since Jan 1, 1980, with no language restrictions. All modes of operative birth, caesarean section and operative vaginal (forceps and vacuum-assisted) births are known to be associated with post-partum infection. The proportion of women who develop an infection without antibiotic prophylaxis is estimated to be 20–25% after a caesarean birth and up to 16% after an operative vaginal birth. A Cochrane systematic review, updated in 2014, identified 95 trials of antibiotic prophylaxis in women giving birth by caesarean section, and showed that the use of prophylactic antibiotics reduced the incidence of wound infection, endometritis, and serious maternal infection by 60–70%. By contrast, a Cochrane review of antibiotic prophylaxis in operative vaginal birth, updated in 2017, identified only one small, low-quality evidence trial, of 393 women reporting only two outcomes: endometritis and maternal length of hospital stay. There was no evidence of a difference in infections between women receiving an antibiotic and those receiving placebo. Recognising the importance of antibiotic stewardship where evidence of benefit is lacking, current WHO and national guidelines therefore explicitly state that routine antibiotic prophylaxis is not recommended for women undergoing operative vaginal birth.

**Added value of this study**

This large multicentre trial was adequately powered to robustly examine the effect of antibiotic prophylaxis after operative vaginal birth. It showed a clear reduction in the proportion of women who had a confirmed or suspected infection after operative vaginal birth in the intervention group, as well as lower proportions of women with perineal wound infection, perineal pain, or perineal wound breakdown after antibiotic prophylaxis, which, to the best of our knowledge, has not previously been shown.

**Implications of all the available evidence**

Antibiotic prophylaxis is effective in preventing up to a half of confirmed or suspected maternal infections during the 6 weeks after operative vaginal birth. These findings suggest that current guidance should be updated to reflect the benefit of routine antibiotic prophylaxis after operative vaginal birth in clinical practice.

## Methods

### Trial design and oversight

The ANODE trial was a multicentre, randomised, blinded, controlled trial done at 27 hospital obstetric units in the UK. The trial protocol has been published previously^18^ and the full protocol is available in the appendix. Women who had undergone forceps or vacuum delivery at 36 weeks or greater gestation, with no indication for ongoing prescription of antibiotics in the post-partum period and no contraindications to prophylactic amoxicillin and clavulanic acid, were randomly assigned (1:1) to receive a single intravenous dose of prophylactic amoxicillin and clavulanic acid or placebo. This trial was approved by the Health Research Authority National Reearch Ethics Service Committee South Central–Hampshire B (study ref: 15/SC/0442).

The trial statisticians did the analysis, prepared the closed reports and attended the closed session of the data monitoring committee meetings. The trial statisticians and the data monitoring committee were masked to trial allocation until the database was locked for the final analysis. No other parties had access to the data presented at the closed data monitoring committee meeting.

A 9-month internal pilot was done and evaluated by the trial steering committee according to the guidelines in the appendix.

### Participants

Women were eligible for inclusion if they were age 16 years or older, willing and able to give informed consent, and had undergone operative vaginal birth at 36 weeks or greater gestation. Women undergoing all types of operative vaginal birth were eligible for inclusion, irrespective of the instrument used, whether rotation was undertaken, or the station of the fetal head at the time of instrument application. They were ineligible if they had any clinical indication for antibiotic administration after delivery, including, but not limited to, confirmed antenatal or intrapartum infection, or third-degree or fourth-degree perineal tears (obstetric anal sphincter injury). Receipt of antenatal or intrapartum antibiotics, such as for prolonged rupture of membranes, was not a reason for exclusion if women had no indication for ongoing antibiotic prescription after delivery. Women who had a known allergy to penicillin or any of the components of amoxicillin and clavulanic acid, or who had a history of anaphylaxis to another β-lactam agent were also excluded.

Women gave written consent to participate, unless there were time or other constraints, in which case they gave verbal consent followed by full written consent before hospital discharge for inclusion of their data in the trial and for participation in the planned follow-up.

### Randomisation and masking

A randomisation list was generated by use of permuted blocks of variable size to ensure balance and unpredictability overall. Pack numbers were added to the randomisation list by an independent trials programmer who liaised directly with the packaging and distribution company. Centres were supplied with sealed, sequentially numbered, indistinguishable packs containing active drug or placebo as designated. Women were randomly assigned (1:1) by the allocation of the next sequentially numbered pack once consent and eligibility were established. The implementation of the allocations was monitored by assessment of whether packs were allocated sequentially as per protocol.

Women, most clinicians (including research midwives and those taking consent), and midwives, nurses, or doctors collecting outcome information were masked to allocation. Clinical staff responsible for preparing and checking the trial drug were not masked to allocation but they had no other role in the trial.

### Procedures

Women in the intervention group received a single dose of intravenous amoxicillin and clavulanic acid (1 g amoxicillin and 200 mg clavulanic acid) as soon as possible and no more than 6 h after giving birth. Women in the placebo group received 20 mL of intravenous sterile 0·9% saline within the same timeframe. The intervention was administered after the women had given birth because of concerns about the effects of prenatal exposure to antibiotics on the infant microbiome.^19^ Unlike other surgical sites, the perineal wound cannot be covered, therefore post-delivery administration of antibiotic increased the length of time that there was a therapeutic level of antibiotic from a single dose, to cover for ongoing contamination for as long as possible.

Outcome information, including information on antibiotic prescription for presumed infection, was collected from medical records at hospital discharge and by a telephone interview at 6 weeks post delivery, after which women were sent a questionnaire for collection of data on secondary outcomes. If women responded to the questionnaire, but not the telephone interview, two investigators masked to allocation independently reviewed their questionnaire responses for any evidence that they had the primary outcome. Clinical data on outcomes, including antibiotic prescription, were collected from medical records or the hospital laboratory at initial discharge and at 6 weeks post delivery, if women indicated they had a possible infection, antibiotic prescription for presumed infection, or attended hospital, or if women did not respond to the telephone interview or questionnaire.

All trial data were entered into electronic case report forms in an OpenClinica database in which participants were identified only by a trial specific number. Women's names and other identifying details were stored in a separate bespoke database linked only by the trial number.

### Outcomes

The primary outcome was confirmed or suspected maternal infection within 6 weeks of delivery, as defined by a new prescription of antibiotics for presumed perineal wound-related infection, endometritis or uterine infection, urinary tract infection with systemic features (pyelonephritis or sepsis) or other systemic infection (clinical sepsis); confirmed systemic infection on culture; or endometritis, as defined by the US Centers for Disease Control and Prevention.^20^ An episode of endometritis required at least one of the following criteria to be met: organisms were cultured from fluid (including amniotic fluid) or tissue from endometrium obtained during an invasive procedure or biopsy, or the woman exhibited at least two of fever (>38°C), abdominal pain, uterine tenderness, or purulent drainage from uterus (with no other recognised cause for the latter three symptoms). The primary outcome was modified during the course of the trial at the request of the data monitoring committee to include only antibiotic prescription for the specific indications listed, instead of antibiotic prescription for any indication, to ensure that the primary outcome only included antibiotic prescriptions for infections potentially preventable by the trial intervention. This protocol amendment was made on Nov 30, 2017.

Secondary outcomes examined were systemic sepsis defined according to modified systemic inflammatory response syndrome criteria for pregnancy,^4^ perineal wound infection defined according to the Public Health England surveillance definition of surgical site infection,^21^ perineal pain, use of pain relief, hospital bed stay until discharge, need for additional perineal care, dyspareunia, ability to sit comfortably to feed the baby, maternal health-related quality of life, breastfeeding, wound breakdown, intervention side-effects, health-care resource use and costs.

The safety reporting window for this trial was from administration of intervention to 6 h post administration or discharge—whichever was sooner. Non-serious adverse events were not routinely recorded because the intervention is a licensed product being given at a standard dose; however adverse events that were part of the study outcomes were recorded in the data collection forms.

All serious adverse events were reported immediately, at least within 24 h; except birth defect or congenital anomaly, hypertensive disorder of pregnancy (eg, pre-eclampsia or eclampsia), or post-partum haemorrhage with onset before the intervention, which were not considered to be causally related to the trial intervention because the events occurred before the trial intervention was administered.

### Statistical analysis

The systematic review^10^ of antibiotic prophylaxis for operative vaginal birth suggested a conservative estimate of infection post birth of 4%. We anticipated an estimated relative risk reduction of 50% in this percentage with antibiotics on the basis of the reduction observed in the antibiotic prophylaxis for caesarean section trials.^7^ To detect such a difference with 90% statistical power at the two-sided 5% level of significance required 1626 women per group. To account for an estimated 5% loss to follow-up, we aimed to recruit 1712 women per group, a total of 3424 women, to the trial.

Women were analysed in the groups into which they were randomly allocated, regardless of intervention received (full analysis dataset; intention-to-treat [ITT] population). Analysis was done according to a prespecified, approved statistical analysis plan (appendix). Women were described with respect to their demographic and clinical characteristics at trial entry. Characteristics of women with incomplete primary outcome data were also described alongside those with complete primary outcome data. Descriptive analyses used n (%) for binary and categorical variables, and mean (SD) or median (IQR, and minimum and maximum values if appropriate) if the data were skewed for continuous variables.

Comparative analyses of binary outcomes used Poisson regression with robust standard errors, and results are presented as unadjusted risk ratios (RR) with associated CI. 95% CIs are presented for analyses of the primary outcome and more stringent 99% CIs are presented for secondary outcomes. No other adjustment was made for multiple testing. Maternal quality of life, assessed by use of EQ-5D-5L scores estimated with the most up-to-date preference-based value set,^22^ were summarised in each group using mean (SD) and compared between groups using mean differences (99% CI). Hospital bed stay until discharge was summarised as the median (IQR) in each group, and compared using the difference in medians (99% CI) and the Wilcoxon rank-sum test.

Four sensitivity analyses were done examining the primary outcome: restricted to women who had not received antibiotics in the 7 days before delivery, to examine whether any masking of a prophylactic effect was occurring by inclusion of pretreated women; excluding women prescribed antibiotics (other than the trial intervention) within the first 24 h after delivery, and who might therefore already have had an infection at the time of administration of the intervention; restricted to women whose primary outcome was obtained between weeks 6 and 10 after delivery to exclude any biases by over-reporting of outcomes from data returned at a later timepoint or under-reporting of outcomes in data returned at an earlier timepoint; and including centre as a random effect. No subgroup analyses were planned; however, we did a post-hoc subgroup analysis of the primary outcome according to mode of birth (forceps or vacuum extraction). More stringent 99% CIs are presented for the estimate of RR for this post-hoc subgroup analysis.

As a further analysis, not included in the original trial protocol, we did a within-trial comparison of health-care resource use and associated costs among women who completed the 6-week post-delivery questionnaire. The perspective of the analysis was that of the UK National Health Service (NHS) and the following categories of resource use were considered: antibiotic use (intervention and new prescriptions), health-care professional visits, outpatient hospital visits, and all-cause hospital readmissions. Sources and associated estimates of unit costs (expressed in 2017/18 GBP) are presented in the appendix. Costs associated to each category of resource use were estimated by multiplying resource use by unit costs. We did a complete case analysis and present mean (SD) of the number of visits or number of days (for readmissions) for each category of resource use and trial group. We also present overall mean (SD) costs at 6 weeks following delivery adding all individual cost categories. Mean differences in resource use and overall costs were calculated and associated 99% parametric CI estimated.

All analyses were done using Stata, version 15.

The trial was overseen by an independent trial steering committee and safety was monitored by an independent data monitoring committee. The data monitoring committee reviewed interim efficacy and safety data at least annually, or more often if appropriate, and advised on the conduct of the trial to the trial steering committee.

This trial is registered with ISRCTN, number 11166984, and is closed to accrual.

### Role of the funding source

ANODE was funded by the National Institute for Health Research HTA Programme (project number 13/96/07). The funder had no role in study design, data collection, data analysis, data interpretation or writing of the report. Authors MK, VC, OR-A, XH, LL, and EJ had full access to all the data in the study and MK had final responsibility for the decision to submit for publication.

## Results

Between March 13, 2016, and June 13, 2018, 3427 women were randomly assigned to treatment: 1719 to amoxicillin and clavulanic acid and 1708 to placebo. Seven women withdrew after allocation; thus, 3420 were included in the primary ITT analysis, 1715 in the amoxicillin and clavulanic acid group and 1705 in the placebo group (figure). The baseline characteristics of the two groups were similar (table 1). 2641 (77%) women were primiparous and 1671 (49%) had induction of labour. 437 (13%) women had ruptured membranes for more than 24 h before they gave birth. 2234 (65%) births were by forceps and 1196 (35%) were by vacuum extraction. The majority of women (3044, 89%) had an episiotomy. Primary outcome data were missing for 195 (6%) women; the characteristics of these women were not significantly different from women for whom primary outcome data were available (appendix).

**Figure:**
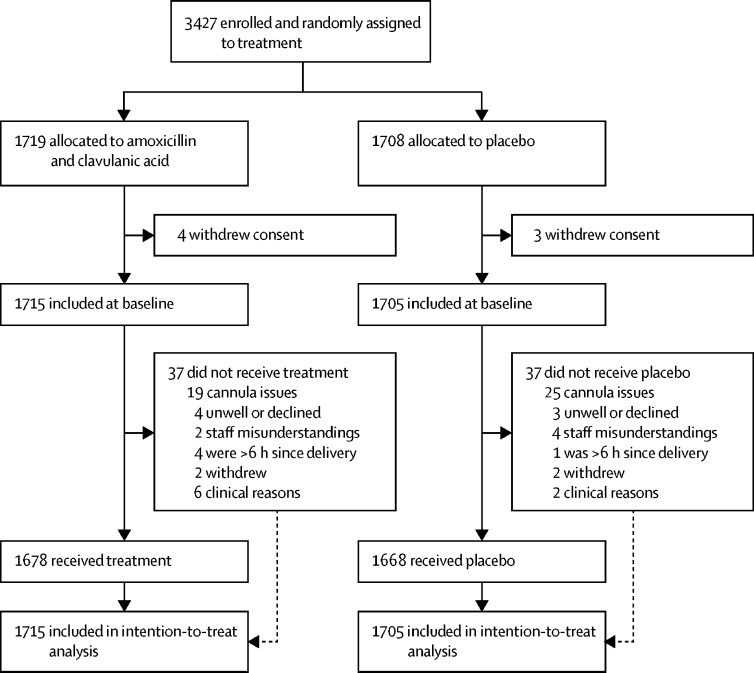
Trial profile Formal screening logs were not kept, so numbers of participants screened and excluded before enrolment are not available.

**Table 1:** Baseline characteristics (intention-to-treat population)

		**Amoxicillin and clavulanic acid (n=1715)**	**Placebo (n=1705)**
Maternal age, years		30·3 (5·37)	30·2 (5·49)
	Missing	0	0
Gestational age at randomisation, weeks		40 (39–41)	40 (39–41)
	36 to <38	136 (8%)	123 (7%)
	38 to <40	568 (33%)	555 (33%)
	40 to <42	964 (56%)	968 (57%)
	≥42	46 (3%)	59 (3%)
	Missing	1	0
Ethnicity			
	White	1436 (84%)	1474 (87%)
	Indian	36 (2%)	34 (2%)
	Pakistani	73 (4%)	54 (3%)
	Bangladeshi	8 (<1%)	14 (1%)
	Black Caribbean	6 (<1%)	8 (<1%)
	Black African	32 (2%)	29 (2%)
	Other	116 (7%)	85 (5%)
	Missing	8	7
Body-mass index at booking, kg/m^2^		25 (22–28)	25 (22–29)
	<18·5	46 (3%)	48 (3%)
	18·5–24·9	851 (51%)	842 (51%)
	25–29·9	460 (28%)	446 (27%)
	30–34·9	207 (12%)	216 (13%)
	35–39·9	74 (4%)	77 (5%)
	≥40	32 (2%)	34 (2%)
	Missing	45	42
Twin pregnancy		11 (1%)	9 (1%)
	Missing	0	0
Any previous pregnancies ≥22 weeks gestation		402 (23%)	373 (22%)
	Missing	1	3
Previous caesarean section		137 (8%)	123 (7%)
	Missing	2	3
Previous episiotomy		147 (9%)	141 (8%)
	Missing	26	25
Previous tear		81 (5%)	80 (5%)
	Missing	24	26
Rupture of membranes before delivery, h		1692 (99%)	1683 (99%)
	<24	1461 (85%)	1466 (86%)
	≥24 to <48	191 (11%)	175 (10%)
	≥48	35 (2%)	36 (2%)
	Unknown	5 (<1%)	6 (<1%)
	Missing	0	0
	Labour induction	819 (48%)	852 (50%)
	Missing	0	0
Actual mode of birth*			
	Spontaneous vaginal	7 (<1%)	3 (<1%)
	Forceps	1086 (63%)	1148 (67%)
	Vacuum extraction	633 (37%)	563 (33%)
	Caesarean section	0	0
	Missing	0	0
Sequential instruments used		77 (4%)	78 (5%)
	Missing	0	0
Reason for instrumental delivery (non-exclusive)			
	Failure to progress	855 (50%)	870 (51%)
	Fetal compromise	861 (50%)	817 (48%)
	Other	134 (8%)	131 (8%)
	Missing	2	0
Episiotomy in current delivery		1519 (89%)	1525 (89%)
	Missing	0	0
Perineal tear in current delivery		493 (29%)	560 (33%)
	Missing	0	0
Perineal wound sutured		1645 (99%)	1665 (100%)
	Missing	54	33
Location of suturing			
	Operating theatre	571 (35%)	588 (35%)
	Delivery ward or room	1074 (65%)	1076 (65%)
	Missing	70	41

Data are mean (SD), n, median (IQR), or n (%).

* Includes 20 sets of twins; 3440 births in total.

74 (2%) women (37 in each group) did not receive their allocated intervention, 44 (59%) because of cannula issues (figure). Women allocated to the intervention group received amoxicillin and clavulanic acid a median of 3·2 h (IQR 2·2–4·5) after they gave birth; 19 (1%) women received the antibiotic more than 6 h after giving birth. Women allocated to the placebo group received saline a median of 3·1 h (IQR 2·1–4·4) after they gave birth; 14 (1%) women received the placebo more than 6 h after giving birth.

The proportion of women who had overall primary outcome events was higher than anticipated (486 [15%] of 3225). A significantly smaller number of women allocated to the amoxicillin and clavulanic acid group had a confirmed or suspected infection (180 [11%] of 1619) than women who were allocated to the placebo group (306 [19%] of 1606; RR 0·58, 95% CI 0·49–0·69; p<0·0001; table 2). The primary outcome was dominated by a new prescription of antibiotics for presumed perineal wound-related infection, endometritis or uterine infection, urinary tract infection with systemic features, or other systemic infection; however, the proportion with confirmed systemic infection on culture was also significantly reduced in the amoxicillin and clavulanic acid group compared with the placebo group (0·6% *vs* 1·5%, respectively; RR 0·44, 95% CI 0·22–0·89; p=0·018).

**Table 2:** Outcomes at 6 weeks post-delivery based on data from telephone follow-up and hospital records (intention-to-treat population)

			**Amoxicillin and clavulanic acid (n=1715)**	**Placebo (n=1705)**	RR*	**p value**
Confirmed or suspected maternal infection			180 (11%)	306 (19%)	0·58 (0·49–0·69)†	<0·0001
	Missing		96	99	NA	NA
	Confirmed systemic infection on culture		11 (1%)	25 (1%)	0·44 (0·22–0·89)†	0·018
		Missing	1	1	NA	NA
	Endometritis		15 (1%)	23 (1%)	0·65 (0·34–1·24)†	0·186
		Missing	1	1	NA	NA
	New prescription of antibiotics with relevant indication		180 (11%)	306 (19%)	0·58 (0·49–0·69)†	<0·0001
		Missing	96	99	NA	NA
Systemic sepsis according to modified SIRS criteria for pregnancy			6 (<1%)	10 (1%)	0·59 (0·16–2·24)‡	0·307
	Missing		9	16	NA	NA
Perineal wound infection						
	Superficial incisional infection		75 (4%)	141 (8%)	0·53 (0·37–0·75)‡	<0·0001
		Missing	3	9	NA	NA
	Deep incisional infection		36 (2%)	77 (5%)	0·46 (0·28–0·77)‡	<0·0001
		Missing	5	11	NA	NA
	Organ or space infection		0	4 (<1%)	0	0·044
		Missing	7	11	NA	NA

Data are n (%), risk ratio (RR; 95% CI), or RR (99% CI). NA=not applicable. SIRS=systemic inflammatory response syndrome.

* Risk in amoxicillin and clavulanic acid group/risk in placebo group.

† 95% CI.

‡ 99% CI.

At 6-weeks post delivery, the proportions of women with perineal infection (table 2), perineal pain, use of pain relief for perineal pain, need for additional perineal care and wound breakdown (table 3), were significantly lower among women allocated to the amoxicillin and clavulanic acid group compared with the placebo group. Women allocated to the amoxicillin and clavulanic acid group were also significantly less likely to report that their perineum was ever too uncomfortable to feed their baby, or to report any primary care physician or home visits or any hospital outpatient visits in relation to concerns about their perineum compared with those allocated placebo (table 3). The incidence of systemic sepsis defined according to modified systemic inflammatory response syndrome criteria for pregnancy (table 2), the proportion of women who were breastfeeding, the proportion of women who had dyspareunia, length of hospital stay to discharge or number of hospital readmissions, or overall maternal quality of life were not different between the two groups (table 3).

**Table 3:** Secondary outcomes at 6 weeks post-delivery based on data from questionnaire (intention-to-treat population)

		**Amoxicillin and clavulanic acid (n=1296)**	**Placebo (n=1297)**	**Effect measure (99% CI)**	**p value**
Perineal pain		592 (46%)	707 (55%)	0·84 (0·76 to 0·93)*	<0·0001
	Missing	0	0	NA	NA
Use of pain relief for perineal pain		99 (8%)	138 (11%)	0·72 (0·52 to 0·99)*	0·0073
	Missing	13	18	NA	NA
Need for additional perineal care		390 (31%)	543 (43%)	0·72 (0·63 to 0·83)*	<0·0001
	Missing	42	38	NA	NA
Wound breakdown		142 (11%)	272 (21%)	0·52 (0·41 to 0·67)*	<0·0001
	Missing	4	7	NA	NA
Dyspareunia†		299 (55%)	280 (54%)	1·01 (0·87 to 1·17)*	0·873
	Missing	5	8	NA	NA
Breastfeeding at 6 weeks		662 (51%)	657 (51%)	1·01 (0·91 to 1·11)*	0·828
	Missing	4	4	NA	NA
Perineum ever too painful or uncomfortable to feed baby		136 (11%)	198 (17%)	0·69 (0·53 to 0·90)*	<0·00025
	Missing	96	98	NA	NA
Hospital bed stay to discharge		1 (1–2)	1 (1–2)	0 (0 to 0)‡	0·318
	Missing	0	0	NA	NA
Any primary care or home visits in relation to perineum		361 (28%)	496 (38%)	0·73 (0·63 to 0·84)*	<0·0001
	Missing	3	5	NA	NA
Any outpatient visits in relation to perineum		95 (7%)	173 (13%)	0·55 (0·40 to 0·75)*	<0·0001
	Missing	5	6	NA	NA
Maternal hospital re-admission		63 (5%)	84 (7%)	0·75 (0·49 to 1·14)*	0·072
	Missing	47	51	NA	NA
Maternal health-related quality of life					
	EQ-5D-5L score	0·935 (0·098)	0·927 (0·111)	0·008 (−0·003 to 0·019)§	0·048
	Missing	16	18	NA	NA

Data are n (%), n, median (IQR), or mean (SD). NA=not applicable. EQ-5D-5L=five-level EuroQol-5D questionnaire.

* Risk ratio (risk in amoxicillin and clavulanic acid group/risk in placebo group).

† Denominator is all women who have attempted intercourse since giving birth (n=544 amoxicillin and clavulanic acid group, n=514 control group).

‡ Difference in medians for hospital bed stay to discharge.

§ Difference in means.

Side-effects of the trial intervention were reported by three women. One woman in the placebo group reported a skin rash and two women in the amoxicillin and clavulanic acid group reported other allergic reactions. One of these allergic reactions was reported as a serious adverse event. Two other serious adverse events were reported, neither was considered causally related to the trial intervention. No women had an anaphylactic reaction.

Results of all sensitivity analyses were not different to those of the main analysis (appendix). The estimate of effect in the post-hoc analysis of the primary outcome according to mode of operative birth was not signiticantly different between the subgroups (p=0·727; appendix).

At 6 weeks post-delivery, women allocated to receive amoxicillin and clavulanic acid used fewer NHS health-care resources compared with women allocated to receive placebo (appendix). The mean difference in all categories of resource use favoured the amoxicillin and clavulanic acid group, with significant mean differences in visits to the general practitioner (mean difference −0·11 visits, 99% CI −0·17 to −0·04), nurse or midwife home visits (−0·18 visits, −0·30 to −0·06), and outpatient hospital visits (−0·14 visits, −0·24 to −0·04). No significant mean differences were detected in the length of stay for all cause hospital readmissions. The total mean costs at 6 weeks following delivery was estimated to be £102·50 (SD £652·40) in the amoxicillin and clavulanic acid group and £155·10 (£497·40) in the placebo group—a mean difference of −£52·60 (99% CI −£115·10 to £9·90).

## Discussion

The ANODE trial showed that women who received a single prophylactic dose of intravenous amoxicillin and clavulanic acid a median of 3 h after operative vaginal delivery were significantly less likely to have a confirmed or suspected maternal infection than women who received placebo. Women receiving antibiotic prophylaxis had a 56% reduction in the risk of confirmed systemic infection on culture compared with women receiving placebo. They were also significantly less likely to experience a range of other secondary outcomes, including perineal wound infection, perineal pain, and perineal wound breakdown. They were less likely to report any primary care physician or home visits or any hospital outpatient visits in relation to concerns about their perineum compared with the placebo group.

The ANODE trial therefore provides evidence of benefit of prophylactic antibiotic administration after operative vaginal birth, with few observed adverse events in relation to the intervention, indicating an urgent need to change current clinical practice to prevent maternal morbidity. Our post-hoc subgroup analysis (appendix) showed that an almost halving of the number of women with an infection was seen both among women who had a forceps birth and women who had vacuum-assisted births, although the proportion of women with confirmed or suspected infection was lower among women who had vacuum-assisted births. The additional resource use analysis (appendix) done for the ANODE trial estimates that for each additional 100 doses of antibiotic used in prophylaxis, 168 treatment doses will be saved, representing a 17% overall reduction in antibiotic use with a policy of universal prophylaxis.

WHO guidelines for the prevention of maternal infection,^9^ and national professional organisational guidelines on operative vaginal delivery in the UK, North America, and Australasia currently do not recommend antibiotic prophylaxis after instrumental vaginal birth.^12^, ^13^, ^14^, ^16^ The proportions of women who have operative vaginal birth are variable; 12% of women have instrumental vaginal birth in the UK;^23^ in the USA the figure is closer to 3–4%^16^, ^24^ and it is estimated to be less than 1% in some low-income settings.^25^ From our results, a high proportion of women—almost one in five—experience an infective complication and this can be reduced by almost half. This equates to prevention of more than 7000 infections annually in the UK with associated perineal complications, and around 5000 per year in the USA with routine use of antibiotic prophylaxis at operative vaginal delivery.

In this trial, we defined suspected or confirmed maternal infection using a composite outcome, including a new prescription of antibiotics for confirmed or suspected infection, which can be interpreted as equating to a clinical diagnosis of infection. This clinical diagnosis of infection drove the overall outcome and resulted in a substantially higher than anticipated percentage of events albeit with an effect size in keeping with our previous assumption, reflecting the high rate of perineal wound infection, which was not measured in the previous trial, and on which we based our anticipated event number. The use of this clinical definition, rather than microbiologically confirmed infection, could be regarded as a limitation; however, we observed a significant decrease in the percentage of microbiologically confirmed systemic infection following culture from a sterile site, which supports the assumption that the findings represent a true decrease in infection, despite our pragmatic primary outcome definition. Our primary outcome definition enhances the generalisability of our findings to low-income and middle-income settings in which microbiological confirmation of infection is difficult or impossible, with a clinical diagnosis of infection the only possible proxy.

We achieved a follow-up of only 76% for the majority of our secondary outcomes, which represents the main limitation of this trial. A greater proportion of women who returned the 6-week questionnaire were primiparous. Their consultation behaviour might have differed from multiparous women, and thus the percentages of some of the reported secondary outcomes might be higher than would be seen in the general population. However, this difference in characteristics between women with and without follow-up data is unlikely to account for the magnitude of difference we observed between the amoxicillin and clavulanic acid group and the placebo group.

We asked sites to administer the intervention as soon as possible, and no later than 6 h, after women had given birth; in practice, it was administered a median of 3 h after women had given birth. It is possible that administration this length of time after women had given birth made the amoxicillin and clavulanic acid less effective than it might have been earlier, given that caesarean section trials suggest that predelivery administration is more effective at preventing wound infection and endometritis. However, the perineal wound, which is highly likely to become contaminated, is different from the caesarean section wound, and later administration might have allowed for a longer duration of protective effect and greater efficacy. Further analyses are needed to investigate the mechanism of effect.

This study shows a high proportion of complications after operative vaginal delivery, not illustrated as clearly in other studies, which generally report proportions of infection lower than 10%. Many observational studies of infections after operative vaginal birth only followed up women until hospital discharge.^8^ The majority of complications we observed occurred after discharge and follow-up to 6 weeks might be regarded as a strength of this trial. Even in the antibiotic group, more than one in every ten women had a postnatal infective complication, which emphasises the importance of ongoing awareness of potential infection and further research to identify ways to reduce this proportion further. Almost half of women reported ongoing perineal pain 6 weeks after giving birth and 11% had a breakdown of their perineal wound. It is unclear whether these effects will continue long term, although other studies have reported ongoing problems in women 6–9 months after a dehisced perineal wound.^26^

This trial was limited to women who were not allergic to penicillin, and used a single antibiotic. However, the results are likely to be comparable if antibiotics with a similar spectrum of activity are used and would therefore be generalisable to women who are allergic to penicillin. This trial was done in a high-income setting, but given the pragmatic nature of the trial and the simple intervention, the findings are likely to be generalisable to low-income and middle-income settings in which intravenous antibiotics are available. It is unclear whether prophylactic antibiotics administered orally would have the same preventive efficacy and this would need further research. The antibiotic was effective when administered a median of 3 h after delivery, but nevertheless 11% of women who received the antibiotic had a confirmed or suspected infection. Further analysis of the mechanism of action of this single dose of antibiotic is needed to investigate whether earlier administration, prenatal administration, or repeated administration is likely to be more effective than the single intravenous dose administered 3 h after women gave birth. Until these analyses are completed, there is no indication for administration of more than a single dose of prophylactic antibiotic, or for predelivery administration. In conclusion, this trial shows clear benefit of a single dose of prophylactic antibiotic after operative vaginal birth, and guidance should be changed to reflect this finding.

**This online publication has been corrected. The corrected version first appeared at thelancet.com on June 13, 2019**

## Data sharing

De-identified participant data will be shared in accordance with the National Perinatal Epidemiology Unit Data Sharing policy. Requests for access to the data will be considered by the National Perinatal Epidemiology Unit Data Sharing committee from the date of publication. Data will be shared after approval of a proposal with investigator approval and completion of a signed data sharing agreement. The trial protocol, statistical analysis plan, and other study documents are also available on request through this route. Access to the data can be requested from general@npeu.ox.ac.uk.
