# Department of Error

**DOI:** 10.1016/S0140-6736(19)31209-7

**Published:** 2019-06-15

*Knight M, Chiocchia V, Partlett C, et al. Prophylactic antibiotics in the prevention of infection after operative vaginal delivery (ANODE): a multicentre randomised controlled trial.* Lancet *2019; published online May 13.* 393: *2395–403*—In this Article, members of the ANODE collaborative group have now been listed at the end of the Article. The Contributors statement has also been corrected to include the contributions of the ANODE collaborative group members. These corrections have been made to the online version as of June 13, 2019, and the printed Article is correct.

