# Supplementary material for: Prophylactic antibiotics in the prevention of infection after operative vaginal delivery (ANODE): a multicentre randomised controlled trial
Source: Lancet. 2019 Jun 15;393(10189):2395–403. doi: 10.1016/S0140-6736(19)30773-1 (PMC6584562; doi:10.1016/S0140-6736(19)30773-1)
Supplement: Supplementary appendix [file mmc1.pdf]

THE LANCET

Supplementary appendix

This appendix formed part of the original submission and has been peer reviewed. We post it as supplied by the authors.

Supplement to: Knight M, Chiocchia V, Partlett C, et al. Prophylactic antibiotics in the prevention of infection after operative vaginal delivery (ANODE): a multicentre randomised controlled trial. *Lancet* 2019; published online May 13. http://dx.doi.org/10.1016/S0140-6736(19)30773-1.

WEB APPENDIX
(ANODE: a multicentre randomised controlled trial of prophylactic ANtibiotics to investigate the prevention of infection following Operative vaginal Delivery)

TABLE OF CONTENTS

ANODE Collaborative Group members 2

Trial Protocol: ANODE: a randomised controlled trial of prophylactic ANtibiotics to investigate the prevention of infection following Operative vaginal DElivery.. 3

ANODE Trial Statistical Analysis Plan 38

Table S1: Source of unit costs for different health care resources 56

Table S2: Comparison of characteristics of women with complete versus missing primary outcome data......... 57

Table S3: Sensitivity analysis: Primary outcome restricted to women who had not received antibiotics in the seven days prior to delivery 58

Table S4: Sensitivity analysis: Primary outcome restricted to women who had not been given antibiotics within 24 hours of delivery.. 59

Table S5: Sensitivity analysis: Primary outcome restricted to women whose primary outcome was obtained from telephone script or questionnaire completed between 6 and 10 weeks post-delivery 60

Table S6: Sensitivity analysis: Primary outcome analysis using centre as random effect................................. 61

Table S7: Post hoc subgroup analysis of composite primary outcome by mode of instrumental delivery.......... 62

Table S8: Mean (SD) healthcare resource use and total cost (UK British pounds 2017/2018) by trial arm and mean differences between trial arms (ITT population).. 63

Table S9: Median and quartiles healthcare resource use and total cost (UK British pounds 2017/2018) by trial arm and mean differences between trial arms 64

Supplementary appendix A1: Evaluation of the ANODE Internal Pilot.. 65

Supplementary appendix A2: Summary of interim and final analysis of the composite primary outcome presented to the DMC.. 67

Table S10: Treatment effect on primary outcome by centre 68

Data Monitoring Committee and Trial Steering Committee members 69

CONFIDENTIAL

ANODE Collaborative Group members

This manuscript is published on behalf of the ANODE Collaborative Group:

Marian Knight, National Perinatal Epidemiology Unit, Nuffield Department of Population Health, University of Oxford
Helen Enderby, lay member, PRIME group.
Derek Tuffnell, Bradford Teaching Hospitals NHS Foundation Trust.
Kim Hinshaw, Sunderland Royal Hospital, Tyne & Wear.
Ranee Thakar, Croydon University Hospital.
Abdul H Sultan, Croydon University Hospital and St George's, University of London.
Julia Sanders, Cardiff University.
Dharmintra Pasupathy, King's College London.
Philip Moore, Birmingham Women's Hospital.
James Gray, Birmingham Children's and Women's Hospitals.
Oliver Rivero-Arias, National Perinatal Epidemiology Unit, Nuffield Department of Population Health, University of Oxford.
Ed Juszczak, NPEU Clinical Trials Unit, National Perinatal Epidemiology Unit, Nuffield Department of Population Health, University of Oxford.
Louise Linsell, NPEU Clinical Trials Unit, National Perinatal Epidemiology Unit, Nuffield Department of Population Health, University of Oxford.
Aethele Khunda, James Cook University Hospital, Middlesbrough.

Trial Protocol: ANODE: a randomised controlled trial of prophylactic ANtibiotics to investigate the prevention of infection following Operative vaginal DElivery

Trial Protocol: ANODE: a randomised controlled trial of prophylactic ANtibiotics to investigate the prevention of infection following Operative vaginal DElivery

Internal Reference Number / Short title: ANODE: prophylactic ANtibiotics for the prevention of infection following Operative DElivery

Ethics Ref: 15/SC/0442

EudraCT Number: 2015-000872-89

ISRCTN11166984

Date and Version No: Version 5.0, 01/12/2017

Chief Investigator: Professor Marian Knight

National Perinatal Epidemiology Unit, Nuffield Department of Population Health, University of Oxford

Tel: 01865 289727

Email: marian.knight@npeu.ox.ac.uk

Sponsor: University of Oxford

Joint Research Office, Block 60, Churchill Hospital, Old Road, Headington, Oxford, OX3 7LE

Funder: National Institute for Health Research Health Technology Assessment programme

Conflicts of interest

None to declare.

TABLE OF CONTENTS

1. KEY TRIAL CONTACTS ... 8
2. SYNOPSIS ... 8
3. TRIAL FLOW CHART ... 11
4. ABBREVIATIONS ... 12
5. BACKGROUND AND RATIONALE ... 14
6. OBJECTIVES AND OUTCOME MEASURES ... 15
7. TRIAL DESIGN ... 16
7.1. Structure and Duration of the Study ... 17
8. PARTICIPANT IDENTIFICATION ... 17
8.1. Trial Participants ... 17
8.2. Inclusion Criteria ... 17
8.3. Exclusion Criteria ... 17
9. TRIAL PROCEDURES ... 18
9.1. Trial Assessments ... 18
9.2. Recruitment ... 19
9.3. Informed Consent ... 20
9.4. Screening and Eligibility Assessment ... 20
9.5. Randomisation, blinding and code-breaking ... 20
9.6. Baseline Assessments ... 21
9.7. Definitions ... 21
9.8. Follow-up Assessments and Data Collection ... 22
9.9. Discontinuation/Withdrawal of Participants from Trial Treatment ... 23
9.10. Definition of End of Trial ... 23
10. INVESTIGATIONAL MEDICINAL PRODUCT (IMP) ... 23
10.1. IMP Description ... 23
10.2. Storage of IMP ... 23
10.3. Accountability of the Trial Intervention ... 23
10.4. Concomitant Medication ... 23
10.5. Post-trial Treatment ... 24
11. SAFETY REPORTING ... 24
11.1. Definitions ... 24
11.2. Causality ... 24
11.3. Procedures for Recording Adverse Events and Reporting Serious Adverse Events ... 25
11.4. Expectedness ... 26
11.5. SUSAR Reporting ... 26
11.6. Safety Monitoring Committee ... 26
11.7. Development Safety Update Reports ... 26
12. STATISTICS ... 26

12.1. The Number of Participants ... 26
12.2. Description of Statistical Methods ... 26
12.3. The Level of Statistical Significance ... 27
12.4. Early Trial Cessation ... 27
12.5. Dealing with Missing Data. ... 27
12.6. Procedures for Reporting any Deviation(s) from the Original Statistical Plan ... 27
13. DATA MANAGEMENT ... 27
13.1. Source Data ... 27
13.2. Access to Data ... 28
13.3. Data Recording and Record Keeping ... 28
14. QUALITY ASSURANCE PROCEDURES ... 28
14.1. Risk Assessment ... 28
14.2. National Registration Systems ... 28
14.3. Site Initiation and Training ... 28
14.4. Data Collection and Processing ... 29
14.5. Central and Site Monitoring ... 29
15. TRIAL GOVERNANCE ... 29
15.1. Site Research and Development Approval ... 29
15.2. Trial Sponsor ... 29
15.3. Co-ordinating Centre ... 29
15.4. Project Management Group ... 29
15.5. Trial Steering Committee ... 29
15.6. Data Monitoring Committee ... 29
16. SERIOUS BREACHES ... 30
17. ETHICAL AND REGULATORY CONSIDERATIONS ... 30
17.1. Declaration of Helsinki ... 30
17.2. Guidelines for Good Clinical Practice ... 30
17.3. Approvals ... 30
17.4. Reporting ... 30
17.5. Participant Confidentiality ... 30
17.6. Expenses and Benefits ... 30
18. FINANCE AND INSURANCE ... 31
18.1. Funding ... 31
18.2. Insurance ... 31
19. PUBLICATION POLICY ... 31
20. REFERENCES ... 32
21. APPENDIX A: PLANNED RECRUITMENT ... 34
22. APPENDIX B: AMENDMENT HISTORY ... 35

1. KEY TRIAL CONTACTS

**Chief Investigator**	Professor Marian Knight<br><br>National Perinatal Epidemiology Unit<br>Nuffield Department of Population Health<br>University of Oxford<br>Old Road Campus<br>Oxford<br>OX3 7LF<br>Phone: 01865 289700<br>Email: marian.knight@npeu.ox.ac.uk<br>Fax: 01865 289701
**Sponsor**	University of Oxford<br><br>Ms Heather House<br>Clinical Trials and Research Governance<br>Joint Research Office<br>Block 60, Churchill Hospital<br>Headington<br>Oxford OX3 7LE<br>Phone: 01865 572224<br>E-mail: ctrg@admin.ox.ac.uk<br>Fax: 01865 572228
**Clinical Trials Unit**	NPEU Clinical Trials Unit<br><br>National Perinatal Epidemiology Unit<br>Nuffield Department of Population Health<br>University of Oxford<br>Old Road Campus<br>Oxford OX3 7LF<br>Phone: 01865 289728<br>Email: ctu@npeu.ox.ac.uk<br>Fax: 01865 289740
**Statistician**	Ms Louise Linsell<br><br>NPEU Clinical Trials Unit<br>National Perinatal Epidemiology Unit<br>Nuffield Department of Population Health<br>University of Oxford<br>Old Road Campus<br>Oxford<br>OX3 7LF<br>Phone: 01865 289700<br>Email: louise.linsell@npeu.ox.ac.uk<br>Fax: 01865 289701

2. SYNOPSIS

**Trial Title**	ANODE: a randomised controlled trial of prophylactic ANtibiotics to investigate the prevention of infection following Operative vaginal DElivery
**Internal ref. no. (or short title)**	ANODE: prophylactic ANtibiotics for the prevention of infection following Operative DElivery

**Clinical Phase**	IV	
**Trial Design**	Multicentre randomised, blinded, placebo-controlled trial	
**Trial Participants**	Women who have had an operative vaginal delivery at 36^{+0} weeks or greater gestation	
**Planned Sample Size**	3,424	
**Treatment duration**	Single dose	
**Follow up duration**	Six weeks	
**Planned Trial Period**	38 months	
	Objectives	Outcome Measures
**Primary**	To compare the incidence of confirmed or suspected maternal infection in the first six weeks after operative vaginal delivery amongst women who have been randomised to receive a prophylactic antibiotic versus those who received a placebo.	**Confirmed or suspected maternal infection within 6 weeks of delivery**, as defined by one of:<br>• A new prescription of antibiotics for presumed perineal wound-related infection, endometritis or uterine infection, urinary tract infection with systemic features or other systemic infection<br>• Confirmed systemic infection on culture<br>• Endometritis as defined by the US Centers for Disease Control and Prevention (Centers for Disease Control and Prevention 2013)
**Secondary**	To investigate the effect of the intervention on various other short-term maternal outcomes, including severe sepsis, perineal wound infection, perineal pain, use of pain relief, hospital bed stay, hospital / GP visits, need for additional perineal care, dyspareunia, ability to sit comfortably to feed the baby, maternal general health, breast feeding, wound breakdown and occurrence of anaphylaxis.	**Systemic sepsis:** defined according to modified SIRS criteria (Waterstone, Bewley et al. 2001, Acosta, Kurinczuk et al. 2013).<br>**Perineal wound infection:** defined according to the Public Health England Surveillance definition of surgical site infection (SSI) (Public Health England (Health Protection Agency) 2013).<br>**Surgical Site infection (perineal):** Identified using the items included in the Public health England "surgical wound healing post discharge questionnaire" (Public Health England (Health Protection Agency) 2013).<br>**Perineal pain/use of pain relief/dyspareunia/ability to sit comfortably to feed the baby/need for additional perineal care/breast feeding:** Identified using standard questions developed for the HOOP study (McCandlish, Bowler et al. 1998) and the PREVIEW study (Ishmail, personal communication).<br>**Maternal general health:** As elicited by the EQ-5D-5L (Herdman, Gudex et al. 2011).

		**Hospital bed stay/Hospital and GP visits/Wound breakdown/antibiotic side effects:** Identified through specific questions included in the maternal questionnaire, to include medications prescribed, critical care admission, hospital inpatient admissions, outpatient visits, and midwife and practice nurse visits.<br>**Hospital admissions and diagnoses at one-year post delivery** identified from linked Hospital Episode Statistics (HES) data or NHS Wales Informatics Service.
**Investigational Medicinal Product(s)**	Co-amoxiclav (active drug) and 0.9% saline (placebo)	
**Formulation, Dose, Route of Administration**	A single intravenous dose (1g amoxicillin/200mg clavulanic acid in 20ml water for injections for active drug, 20ml 0.9% saline for placebo)	

3. TRIAL FLOW CHART

ANODE: prophylactic ANtibiotics for the prevention of infection following Operative DElivery

Eligibility criteria

- Willing and able to give informed consent for participation in the trial
- Women ≥ 36^{+0} weeks of gestation at delivery
- Aged 16 years or above
- Has undergone operative vaginal delivery

Exclusion criteria

- Clinical indication for ongoing antibiotic administration post-delivery e.g. due to confirmed antenatal infection, 3rd or 4th degree tears
- Known allergy to penicillin or to any of the components of co-amoxiclav, as documented in hospital notes
- History of anaphylaxis (a severe hypersensitivity reaction) to another B-lactam agent (e.g. cephalosporin, carbapenem or monobactam), as documented in hospital notes

Randomisation (1:1 allocation ratio)

Sequentially numbered indistinguishable sealed boxes

Active intervention

Single dose of intravenous co-amoxiclav (1g amoxycillin/200mg clavulanic acid)

OR

Placebo control

Placebo (0.9% normal saline)

Primary outcome

Confirmed or suspected maternal infection within 6 weeks of delivery as defined by one of:

- A new prescription of antibiotics for presumed perineal wound-related infection, endometritis or uterine infection, urinary tract infection with systemic features or other systemic infection
- Confirmed systemic infection on culture
- Endometritis as defined by the US Centers for Disease Control and Prevention (Centers for Disease Control and Prevention 2013)

Secondary outcomes

(within 6 weeks of delivery)

- Systemic sepsis
- Perineal wound infection
- Surgical Site infection (perineal)
- Perineal pain/use of pain relief/dyspareunia/ability to sit comfortably to feed the baby/need for additional perineal care/breast feeding
- Maternal general health
- Hospital bed stay/hospital and GP visits/wound breakdown/antibiotic side effects/informal care

4. ABBREVIATIONS

AE	Adverse event
AR	Adverse Reaction
ARR	Absolute Risk Reduction
BERC	Blinded Endpoint Review Committee
CI	Chief Investigator
CIG	Co-Investigator Group
CRN	Clinical Research Network
CT	Clinical Trials
CTA	Clinical Trials Authorisation
CTRG	Clinical Trials and Research Governance Office, University of Oxford
DCF	Data Collection Form
DMC	Data Monitoring Committee
DSUR	Development Safety Update Report
eCRF	Electronic Case Report Form
EDD	Expected Date of Delivery
FTE	Full Time Equivalent
GCP	Good Clinical Practice
GP	General Practitioner
HSCIC	Health and Social Care Information Centre
HTA	Health Technology Assessment
IB	Investigator Brochure
ICF	Informed Consent Form
ICH	International Conference on Harmonisation
IMP	Investigational Medicinal Product
ITT	Intention-To-Treat
LRM	Local Research Midwife
MHRA	Medicines and Healthcare products Regulatory Agency
NHS	National Health Service
NICE	National Institute for Health and Care Excellence
NIHR	National Institute for Health Research
NPEU CTU	National Perinatal Epidemiology Unit Clinical Trials Unit
NRES	National Research Ethics Service
PI	Principal Investigator
PIL	Participant/ Patient Information Leaflet

PMG	Project Management Group
QR	Quick Response
R&D	NHS Trust Research and Development Department
RCOG	Royal College of Obstetricians and Gynaecologists
REC	Research Ethics Committee
RR	Risk Ratio
SAE	Serious Adverse Event
SAR	Serious Adverse Reaction
SMPC	Summary of Medicinal Product Characteristics
SOP	Standard Operating Procedure
SUSAR	Suspected Unexpected Serious Adverse Reactions
TMF	Trial Master File
TSC	Trial Steering Committee

5. BACKGROUND AND RATIONALE

Sepsis is now the most important cause of direct maternal death in the UK (Lewis, Cantwell et al. 2011). In addition to every maternal death, an estimated 50 women have severe sepsis (requiring level 2 or 3 critical care) but survive (Acosta, Kurinczuk et al. 2014). An increased risk of sepsis in association with caesarean section delivery has been recognised for many years (Declercq, Barger et al. 2007), and NICE guidance recommends the use of prophylactic antibiotics at all caesarean deliveries (National Institute for Health and Clinical Excellence 2011), based on substantial randomised controlled trial evidence of effectiveness (Smaill and Gyte 2010). Studies conducted both in the UK and US, have documented an additional risk associated with operative vaginal delivery (Acosta, Bhattacharya et al. 2012, Acosta, Knight et al. 2013, Acosta, Kurinczuk et al. 2014), and particularly in relation to Group A streptococcal infection, the leading and most severe cause of maternal infection (Lewis, Cantwell et al. 2011, Acosta, Kurinczuk et al. 2014). A Cochrane review, updated in 2012, has identified only one small previous trial of prophylactic antibiotics following operative vaginal delivery, including a total of 393 women, with a relative risk of 0.07 (95% confidence interval 0.00 to 1.21) for postpartum infection (Liabsuetrakul, Choobun et al. 2004), and given the small study size and extreme result, recommends that further robust evidence is needed.

Further work suggests that the burden of localised infection following operative vaginal delivery is also significant (Johnson, Thakar et al. 2012), with more than 10% of women experiencing symptoms of perineal wound infection in the three weeks following delivery. Women involved in prioritising childbirth related perineal trauma outcomes have rated “fear of perineal infection” as the most important outcome they are concerned about in the first few weeks after childbirth related perineal trauma (Perkins, Tothill et al. 2008).

Latest figures show that approximately 13% of women have an operative vaginal (forceps or ventouse) delivery in England, representing a significant burden of potentially preventable morbidity (Health and Social Care Information Centre 2012). Current National Institute for Health and Care Excellence (NICE) guidelines for Intrapartum Care make no reference to prophylactic antibiotics following instrumental delivery (National Institute for Health and Clinical Excellence 2007). Royal College of Obstetricians and Gynaecologists (RCOG) Guidance on Operative Vaginal Delivery (Bahl, Strachan et al. 2011) states that there are insufficient data to justify the use of prophylactic antibiotics in operative vaginal delivery, referencing the Cochrane review identified above. RCOG guidance on Bacterial Sepsis following Pregnancy does not identify operative vaginal delivery as a risk factor for postpartum infection (Morgan, Hughes et al. 2012) and lack of awareness of the associated risk may contribute to a delay in diagnosis. Evidence suggests that progression to severe sepsis following delivery, particularly in association with group A streptococcal infection, can be very rapid (Lewis, Cantwell et al. 2011, Acosta, Kurinczuk et al. 2014). This emphasises the importance of urgent investigation of potential prophylactic measures.

Thirteen percent of women in the UK undergo forceps or ventouse deliveries (Health and Social Care Information Centre 2012), an estimated 104,000 women annually. The conservatively estimated incidence of maternal infection following operative vaginal delivery is 4%, based on the one previous trial (Liabsuetrakul, Choobun et al. 2004), resulting in an estimated 4,160 women potentially having an infection after instrumental delivery. Of these women, around 200 will be diagnosed with severe sepsis (Acosta, Bhattacharya et al. 2012), and up to four may die from their infection (Lewis, Cantwell et al. 2011, Acosta, Kurinczuk et al. 2014). There is therefore considerable scope for direct patient benefit from an effective preventive strategy.

The intervention being assessed is a single dose of intravenous co-amoxiclav (1g amoxicillin/200mg clavulanic acid) following delivery, versus a placebo (0.9% normal saline).

Recent recommendations suggest that antibiotic prophylaxis for caesarean section should be given prior to delivery. This trial specifically investigates the use of antibiotic prophylaxis **after** operative vaginal delivery of the infant for the following reasons:

a) There are increasing concerns about the risks of prenatal exposure to antibiotics, with known associations with necrotising enterocolitis (European Centre for Disease Control and Prevention 2011) and cerebral palsy (McCulloch, Altman et al. 2009) amongst the children of women managed with antibiotics for suspected preterm labour. Use of antibiotics in the third trimester has also been associated with an increased risk of

asthma in early childhood (Stensballe, Simonsen et al. 2013), and the potential for antibiotics to alter the infant microbiome and thus have long term impacts on other disease states is also increasingly being recognised (Gulmezoglu and Duley 1998).

b) The major difference between the episiotomy wound and the caesarean section wound is the fact that there is ongoing contamination of the surgical field. Thus, with caesarean section as soon as the operation is completed and a wound dressing applied, the major risk of infection is over. In contrast, an episiotomy wound is impossible to cover and therefore our rationale is to actually increase the length of time that there would be therapeutic levels of antibiotic from a single dose by giving it post-delivery, to cover for ongoing contamination for as long as possible.

c) There have been several cases of anaphylaxis relating to antibiotics given prophylactically for caesarean delivery identified in an ongoing NIHR funded study (M Knight, personal communication). Although the incidence is extremely low, this is of concern particularly with antenatal administration when there is the potential for fetal compromise.

Because of concerns over prenatal exposure to co-amoxiclav, prophylaxis at caesarean delivery has moved towards the use of cephalosporins. However, there are several reasons why co-amoxiclav is preferable to cephalosporins as prophylaxis at operative vaginal delivery and hence this study will investigate the use of co-amoxiclav:

a) Co-amoxiclav has a wider spectrum of activity (encompassing anaerobes and enterococci), which is important in view of the likelihood of perineal contamination with bowel flora and the association of anaerobic bacteria with perineal wound breakdown;

b) Amoxicillin is up to 10-fold more active than cefuroxime against group A streptococci (GAS). GAS is associated with very severe, rapidly progressive postnatal infection and adequate coverage against this organism is essential. We plan to administer prophylaxis after delivery of the baby, thus avoiding any drug-related risk of necrotising enterocolitis;

c) Department of Health and Public Health England guidance on *Clostridium difficile* advises avoidance of use of cephalosporins. Many hospitals have thus relegated cefuroxime from first-line use on the basis of this guidance. A cefuroxime-based regimen is therefore unlikely to be acceptable to many hospitals and would require arrangements for ensuring stocks of different antibiotics.

d) Co-amoxiclav is less likely to select for antibiotic resistances (e.g. MRSA, ESBL- and AmpC-producing Gram-negative bacteria) (Chief Medical Officer 2013). Cephalosporins are associated with selection of a number of antibiotic resistances (as well as C. Difficile), most notoriously MRSA and ESBL-producing Gram-negative bacteria. Also, in neonates, cephalosporins have been associated with an increased risk of candidiasis and there is a theoretical risk of the same in women.

6. OBJECTIVES AND OUTCOME MEASURES

**Objectives**	**Outcome Measures**	**Timepoint(s) of evaluation of this outcome measure**
**Primary Objective**<br>To compare the incidence of confirmed or suspected maternal infection in the first six weeks after operative vaginal delivery amongst women who have been randomised to receive a prophylactic antibiotic versus those who received placebo.	The primary outcome will be **confirmed or suspected maternal infection within 6 weeks of delivery**, as defined by one of:<br>• A new prescription of antibiotics for presumed perineal wound-related infection, endometritis or uterine infection, urinary tract infection with systemic features or other systemic infection<br>• Confirmed systemic infection on culture<br>• Endometritis as defined by the US Centers for Disease Control and	At 6 weeks post-delivery by telephone interview with a research midwife

	Prevention (Centers for Disease Control and Prevention 2013)	
**Secondary Objectives**<br>To investigate the effect of the intervention on various other maternal outcomes, including severe sepsis, perineal wound infection, perineal pain, use of pain relief, hospital bed stay, hospital / GP visits, need for additional perineal care, dyspareunia, ability to sit comfortably to feed the baby, maternal general health, breast feeding, wound breakdown and occurrence of anaphylaxis.	**Systemic sepsis:** defined according to modified SIRS criteria (Waterstone, Bewley et al. 2001, Acosta, Kurinczuk et al. 2013).<br>**Perineal wound infection:** defined according to the Public Health England Surveillance definition of surgical site infection (SSI) (Public Health England (Health Protection Agency) 2013).<br>**Surgical Site infection (perineal):** Identified using the items included in the Public health England "surgical wound healing post discharge questionnaire" (Public Health England (Health Protection Agency) 2013).<br>**Perineal pain/use of pain relief/dyspareunia/ability to sit comfortably to feed the baby/need for additional perineal care/breast feeding:** Identified using standard questions developed for the HOOP study (McCandlish, Bowler et al. 1998) and the PREVIEW study (Ishmail, personal communication).<br>**Maternal general health:** As elicited by the EQ-5D-5L (Herdman, Gudex et al. 2011).<br>**Hospital bed stay/Hospital and GP visits/Wound breakdown/antibiotic side effects:** Identified through specific questions included in the maternal questionnaire, to include medications prescribed, critical care admission, hospital inpatient admissions, outpatient visits, and midwife and practice nurse visits.<br>**Hospital admissions and diagnoses at one-year post delivery** identified from linked Hospital Episode Statistics (HES) data or NHS Wales Informatics Service.	A postal or online questionnaire (as preferred by each woman) at six weeks post-delivery, following initial telephone interview.<br><br>Clinical data collection from the woman's medical records or hospital laboratory at six weeks post-delivery if the initial telephone interview indicates that the woman has been admitted, or had samples sent for culture.<br><br>Data from linked information contained within Hospital Episode Statistics or NHS Wales Informatics Service.

7. TRIAL DESIGN

A multicentre, randomised, blinded, placebo-controlled trial to investigate whether a single dose of prophylactic antibiotic following operative vaginal delivery is clinically effective for preventing confirmed or suspected maternal infection.

Women who have undergone forceps or ventouse delivery at 36^{+0} weeks or greater gestation, with no indication for ongoing prescription of antibiotics in the postpartum period and no contra-indications to prophylactic co-amoxiclav, will be randomised to receive a single intravenous dose of prophylactic co-amoxiclav or placebo.

The research midwife, most clinicians and the women will remain blind to allocation (note that the research midwife will be collecting outcomes information). The people responsible for preparing and checking the trial drug who may be, for example, a doctor, midwife, nurse, Operating Department Practitioner (ODP) or other healthcare professional (centre-dependant), will be the only people not blinded to allocation (these people will not be involved in the collection of outcomes information).

Outcome information will be collected by a single telephone interview and questionnaire, with clinical data collection from medical records or the hospital laboratory if necessary, at six weeks post-delivery. There will be no further follow-up, but participants will be asked for permission to link their records to Hospital Episode Statistics or NHS Wales Informatics Service in order to assess outcomes at one year.

The trial design and schedule of events are summarised in sections 3 and 9.1.

7.1. Structure and Duration of the Study

The trial aims to recruit 3,424 participants from 14 centres in the UK over a period of 26 months (Appendix A). An initial nine month internal pilot study will be undertaken to test whether the components and processes of the study will work together and run smoothly. Projections suggest that approximately 1,128 women could be recruited in that time. Data collected from the internal pilot phase will be included in the final analysis.

The decision to progress with the main trial will be based on efficacy, safety, and logistics and will be made in consultation with the TSC and the funder. Stop/go criteria upon which a decision will be made will be established prior to the start of the internal pilot phase. Should a decision be made not to progress to the main phase, a report on the internal pilot phase will be submitted for publication according to the publication policy.

The total duration of the project is estimated to be 44 months:

Pre-trial: Obtain Research Ethics Committee approval; establish TSC and DMC; recruit trial staff.
Months 1-11: Obtain R&D approvals and set-up study sites; train local personnel in trial procedures.
Months 12-20: 9 months internal pilot study.
Month 21-38: Main trial (post successful pilot study), recruitment in study sites and data collection.
Month 39: Completion of follow-up.
Months 40-44: Analysis, reporting and dissemination of results.

8. PARTICIPANT IDENTIFICATION

8.1. Trial Participants

Women who have undergone operative vaginal delivery at 36^{+0} weeks or greater gestation, with no indication for ongoing prescription of antibiotics in the postpartum period and no contra-indications to prophylactic co-amoxiclav.

8.2. Inclusion Criteria

- Women aged 16 years or above, willing and able to give informed consent.
- Women who have had an operative vaginal delivery at 36^{+0} weeks or greater gestation.

8.3. Exclusion Criteria

Women may not enter the trial if ANY of the following apply:

- Clinical indication for **ongoing** antibiotic administration post-delivery e.g. due to confirmed antenatal infection, 3rd or 4th degree tears. Note that receiving antenatal antibiotics e.g. for maternal Group B Streptococcal carriage or prolonged rupture of membranes, is not a reason for exclusion if there is no indication for ongoing antibiotic prescription post-delivery.
- Known allergy to penicillin or to any of the components of co-amoxiclav, as documented in hospital notes.
- History of anaphylaxis (a severe hypersensitivity reaction) to another β-lactam agent (e.g. cephalosporin, carbapenem or monobactam), as documented in hospital notes.

9. TRIAL PROCEDURES

9.1. Trial Assessments

Procedure	Eligibility screening	Trial Entry and drug administration (day 1)	Up to 6 hours after trial drug was administered	6 weeks of post-delivery
Demography		✓		
Confirmation of Eligibility	✓			
Consent		✓		
Randomisation		✓		
Co-amoxiclav/ Placebo Dosing[1]		✓		
SAEs		✓	✓	
Concomitant Medication[2]		✓	✓	
6 week telephone interview				✓
6 week Mother's Questionnaire				✓

1 Initial trial drug administrations to be given as soon as possible after randomisation.

2 Concomitant medications to be recorded only in relation to SAEs. In the event of an SAE all concomitant medication, from admission to labour ward to time of event, must be recorded on the SAE form.

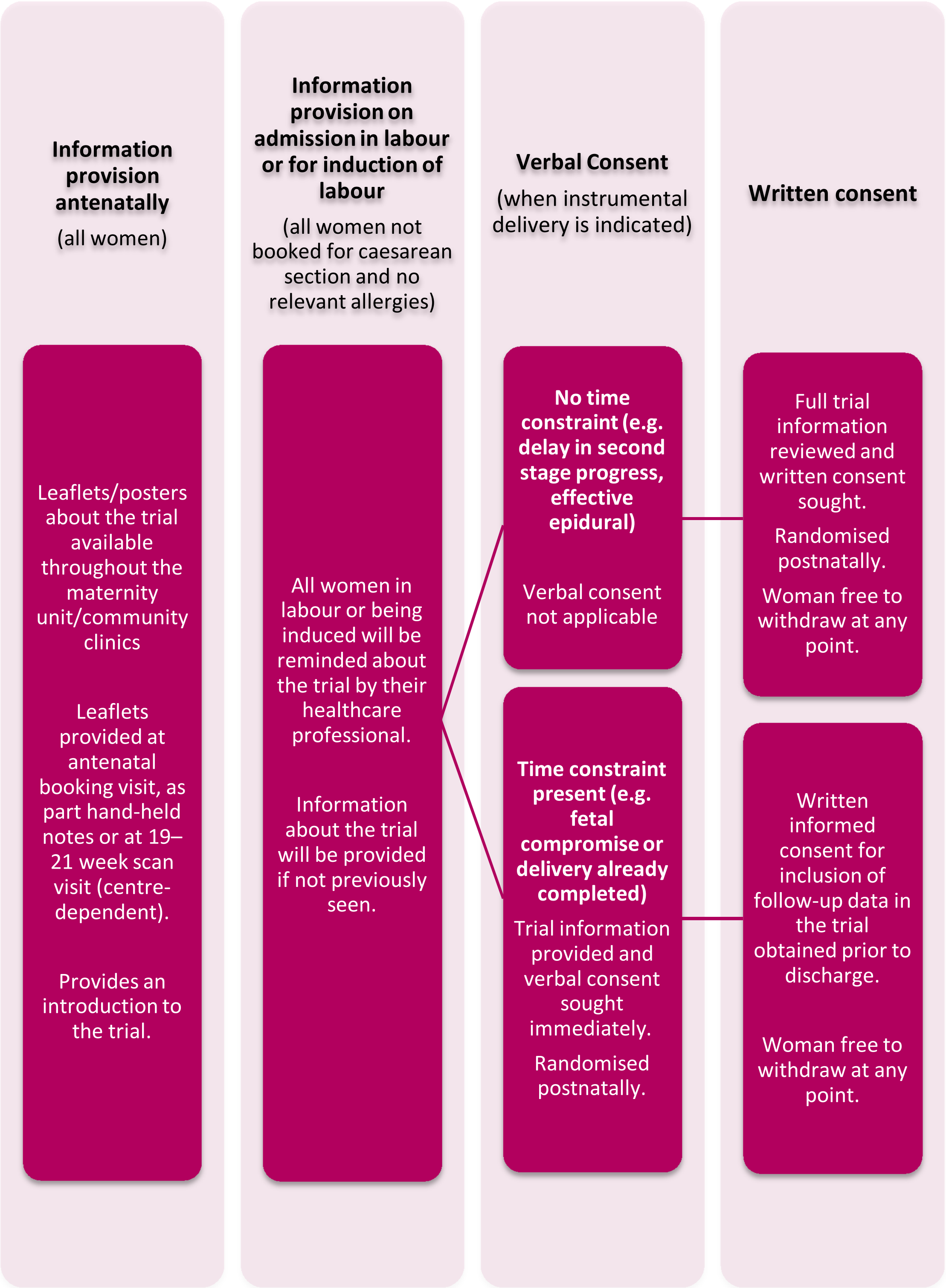

Figure 1: Consent and randomisation processes

9.2. Recruitment

Information about the trial will be widely available throughout the maternity unit and community clinics in the form of posters and leaflets (with QR codes to the trial website). All women at participating centres will be provided with written information about the trial during their pregnancy, for example, at their antenatal booking visit, as part of their hand-held notes or at their 19–21 week scan visit (centre-dependent).

On admission, all women in labour or admitted for induction will be reminded about the trial by their healthcare professional. Information about the trial will be provided if not previously seen. After the clinical decision for operative vaginal delivery is made and the woman or her representative has given consent for operative vaginal delivery, the woman will be approached by her midwife, obstetrician or anaesthetist as described in section 9.3.

9.3. Informed Consent

Written and verbal versions of the Participant Information and Informed Consent will be prepared detailing no less than: the exact nature of the trial; what it will involve for the participant; the implications and constraints of the protocol; the known side effects and any risks involved in taking part. It will be clearly stated that the participant is free to withdraw from the trial at any time for any reason without prejudice to future care, and with no obligation to give the reason for withdrawal.

The following approaches will be used by the woman's midwife, obstetrician or anaesthetist to obtain informed consent, depending on the clinical circumstances (figure 1):

1. Where there is no time constraint (e.g. in cases of operative vaginal delivery for delayed second stage progress), the healthcare professional will discuss the trial with the woman and provide her with the Participant Information leaflet. If she is happy to join the trial informed written consent will be obtained.
2. Where there is a time or other constraint (e.g. in cases of operative vaginal delivery for suspected fetal compromise or delivery is already completed), women will be approached to give verbal consent. It is possible that urgent deliveries are associated with a lower standard of asepsis, and so it is particularly important that these women are able to participate in the trial. Information about the trial will have been available to all women prior to having been admitted to hospital in labour or for induction. If the attending obstetrician or midwife feels it is appropriate, the woman will be provided with verbal information about the trial and asked if she is willing to participate, in principle; if she agrees, she will be randomised. If she does not give verbal consent, she will not be recruited into the trial. Verbal consent will be documented by the clinician recruiting the woman and countersigned by a witness. All women enrolled under this procedure will be approached before discharge by study midwives to give full written consent for inclusion of their data in the trial and for participation in the planned follow-up.

The woman must personally sign and date the latest approved version of the Informed Consent form.

Where there are no time constraints the woman will have the opportunity to ask questions of the care team and other independent parties to decide whether she participates in the trial. Written Informed Consent will be obtained by means of participant dated signature and dated signature of the person who presented and obtained the Informed Consent. The person who obtained the consent must be suitably qualified and experienced, and have been authorised to do so by the Chief/Principal Investigator. A copy of the signed Informed Consent will be given to the woman, a copy placed in her medical records and a copy retained at the trial site. The original signed form will be sent to the coordinating centre.

9.4. Screening and Eligibility Assessment

Women will be assessed for eligibility before and after the operative vaginal delivery. The screening procedure will include assessment of gestational age (obtained from clinical records) and medical history to assess eligibility.

9.5. Randomisation, blinding and code-breaking

A randomisation list will be generated by the Senior Trials Statistician at the NPEU-CTU using permuted blocks of variable size to ensure balance and unpredictability overall. Pack numbers will be added by the Senior Trials Programmer at the NPEU-CTU, who will liaise directly with the packaging and distribution company. The Senior Trial Programmer will be custodian of the complete randomisation schedule and monitor the implementation of the allocations. Centres will be supplied with sealed sequentially numbered indistinguishable packs containing active drug or placebo (saline solution), as designated. Women will be randomised by the allocation of the next sequentially numbered pack once consent and eligibility are established.

An emergency code-breaking procedure will not be required; as only a single dose of co-amoxiclav will be administered there is no need to code-break if further antibiotics are required. If a woman was to have an anaphylactic reaction she would be treated as if she has been given the active drug.

9.6. Baseline Assessments

For eligible women, clinical details will be collected at trial entry (randomisation). This will include details to confirm eligibility including the woman's age, gestational age, mode of delivery and confirmation of written or verbal consent.

9.7. Definitions

Primary outcome:

Confirmed or suspected maternal infection within 6 weeks of delivery, as defined by one of:

- A new prescription of antibiotics for presumed perineal wound-related infection, endometritis or uterine infection, urinary tract infection with systemic features or other systemic infection
- Confirmed systemic infection on culture
- Endometritis as defined by the US Centers for Disease Control and Prevention (Centers for Disease Control and Prevention 2013)

An episode of endometritis requires meeting at least one of the following criteria:
1. Organisms are cultured from fluid (including amniotic fluid) or tissue from endometrium obtained during an invasive procedure or biopsy.
2. Woman exhibits at least two of the following signs or symptoms: fever (>38°C), abdominal pain*, uterine tenderness*, or purulent drainage from uterus*.
* With no other recognised cause

Secondary outcomes (within 6 weeks of delivery):

Systemic sepsis: defined according to modified SIRS criteria for pregnancy used in previous population-based surveillance studies (Waterstone, Bewley et al. 2001, Acosta, Kurinczuk et al. 2013), namely
1. Any woman dying from infection or suspected infection
2. Any woman requiring level 2 or level 3 critical care (or obstetric HDU type care) due to severe sepsis or suspected severe sepsis
3. A clinical diagnosis of severe sepsis (two or more of the following):
   a. Temperature >38°C or <36°C measured on two occasions at least four hours apart
   b. Heart rate >100 beats/minute measured on two occasions at least four hours apart
   c. Respiratory rate >20/minute measured on two occasions at least four hours apart
   d. White cell count $>17x10^9/L$ or $<4x10^9/L$ or with >10% immature band forms, measured on two separate occasions.

Perineal wound infection: defined according to the Public Health England Surveillance definition of surgical site infection (SSI) (Public Health England (Health Protection Agency) 2013), namely

Superficial Incisional Infection: SSI that occurs within 30 days of surgery, involves only the skin or subcutaneous tissue of the incision and meets at least one of the following criteria:

1. Purulent drainage from superficial incision
2. Culture of organisms and pus cells present in: fluid/tissue from superficial incision or wound swab from superficial incision
3. At least two symptoms of inflammation: pain, tenderness, localised swelling, redness, heat

AND EITHER: 1) incision deliberately opened to manage infection
OR 2) clinician's diagnosis of superficial SSI

Deep Incisional Infection: SSI involving the deep tissues (i.e. fascial & muscle layers), within 30 days of surgery (or 1 year if an implant is in place) and the infection appears to be related to the surgical procedure and meets at least one of the following criteria:

1. Purulent drainage from deep incision (not organ space)
2. Organisms from culture and pus cells present in: fluid/tissue from deep incision or wound swab from deep incision
3. Deep incision dehisces or deliberately opened and patient has at least one symptom of: fever or localised pain/tenderness
4. Abscess or other evidence of infection in deep incision: re-operation / histopathology / radiology
5. Clinician's diagnosis of deep incisional SSI

Organ/space Infection: SSI involving the organ/space (other than the incision) opened or manipulated during the surgical procedure, that occurs within 30 days of surgery and the infection appears to be related to the surgical procedure & meets at least one of the following criteria:

1. Purulent drainage from drain (through stab wound) into organ space
2. Organisms from culture and pus cells present in: fluid or tissue from organ/space or swab from organ/space
3. Abscess or other evidence of infection in organ/space: re-operation I histopathology I radiology
4. Clinician's diagnosis of organ/space infection

9.8. Follow-up Assessments and Data Collection

Data will be collected at:

1. Hospital discharge after delivery by extraction of information from the woman's clinical records by the research midwife.
2. 6 weeks post-delivery by telephone interview with a research midwife to obtain information on the primary outcome, following which each woman will be sent a postal or online questionnaire (as preferred by each woman) for collection of data on secondary outcomes.

Any concerns arising from the responses on the follow-up questionnaire will be referred to the CI and actioned appropriately. Text reminders for completion will be sent as appropriate, with the option for telephone completion in the event of a delayed response to ensure a high response rate. Information about any hospital readmissions will be collected from hospital records by the research midwife.

Data on the primary outcome will be collected through specific tailored questionnaires. In addition, case records of participating women will be flagged to allow for additional capture of data relevant to the primary outcome at readmission to hospital, if this occurs. Basic demographic, medical and obstetric details will be collected on all women, including details of any antibiotic treatment in the seven days before delivery.

Data on maternal anaphylaxis will be collected up until hospital discharge. Data on other secondary outcomes will be collected at 6 weeks post-delivery using standard instruments where possible as detailed below.

Surgical Site infection (perineal): Identified using the items included in the Public health England "surgical wound healing post discharge questionnaire" (Public Health England (Health Protection Agency) 2013).
Perineal pain/use of pain relief/dyspareunia/ability to sit comfortably to feed the baby/need for additional perineal care/breast feeding: Identified using standard questions developed for the HOOP study (McCandlish, Bowler et al. 1998) and the PREVIEW study (Ishmail, personal communication).
Maternal general health: As elicited by the EQ-5D-5L (Herdman, Gudex et al. 2011).
Hospital bed stay/Hospital and GP visits/Wound breakdown/antibiotic side effects: Identified through specific questions included in the maternal questionnaire, to include medications prescribed, critical care admission, hospital inpatient admissions, outpatient visits, and midwife and practice nurse visits. All side effects of the IMP will be recorded. Known side effects of the IMP examined will be as follows:
Common side effects (affecting more than 1 in every 100 women): thrush (candida), diarrhoea.

Uncommon side effects (affecting more than 1 in every 1,000 women): urticarial rash, itching, nausea, vomiting, indigestion, dizziness, headache, altered liver enzymes.
Rare side effects (affecting more than 1 in every 10,000 women): erythema multiforme, thrombophlebitis at the injection site, thrombocytopenia, leucopenia.
Frequency unknown: Anaphylaxis.

In order to capture any additional related health outcomes after the 6-weeks post-delivery, we would like to extract hospital inpatients, critical care, outpatients, and A&E information from Hospital Episode Statistics (HES) or NHS Wales Informatics Service up to 1-year follow-up for all trial participants. Explicit consent for this will be sought.

9.9. Discontinuation/Withdrawal of Participants from Trial Treatment

Each woman has the right to withdraw from the trial follow-up at any time following their single dose of antibiotic. Unless we obtain consent to continue to use data at withdrawal, the data for women withdrawing from the trial will be excluded from future analyses, with the exception of safety analyses (i.e. antibiotic side-effects).

The reason for withdrawal will be recorded in the eCRF, if a reason is given.

9.10. Definition of End of Trial

The end of trial is the date when the database is locked.

10. INVESTIGATIONAL MEDICINAL PRODUCT (IMP)

This trial is classified as a type A Clinical Trial of an IMP.

10.1. IMP Description

Trial treatment: a single dose of intravenous co-amoxiclav (1g amoxicillin/200mg clavulanic acid)
Placebo: a single dose of intravenous sterile saline.

Co-amoxiclav 1,000 mg/200 mg powder for solution for injection is supplied as bottles of sterile powder for making up as an injection reconstituted with sterile water for injection, also supplied.

Placebo (0.9% saline) will be supplied as 20ml single use vials of clear liquid. Reconstitution is not required.

10.2. Storage of IMP

A stock of packs will be stored centrally on the delivery suite at room temperature for immediate use. Normal hospital policy with regards to monitoring of storage of co-amoxiclav will apply.

10.3. Accountability of the Trial Intervention

Drug packs will be allocated by selecting the lowest sequentially numbered indistinguishable box available within the recruiting centre. Pack use will be recorded by the recruiting site and reviewed by NPEU CTU. The trial intervention consists of a single intravenous dose of antibiotic or placebo. A record of individual administrations will be kept and the timing of administering the trial intervention will be recorded in the eCRF.

10.4. Concomitant Medication

There are no contra-indicated concomitant medications. Current clinical protocols regarding drug administration will not be altered by this trial (apart from the additional trial medication). All concomitant medications will be recorded in the event of an immediately reportable Serious Adverse Event.

10.5. Post-trial Treatment

There will be no provision of the antibiotic beyond the trial period as it is given as a single dose only.

11. SAFETY REPORTING

11.1. Definitions

Adverse Event (AE)	Any untoward medical occurrence in a participant to whom a medicinal product has been administered, including occurrences which are not necessarily caused by or related to that product.
Adverse Reaction (AR)	An untoward and unintended response in a participant to an investigational medicinal product which is related to any dose administered to that participant.<br>The phrase "response to an investigational medicinal product" means that a causal relationship between a trial medication and an AE is at least a reasonable possibility, i.e. the relationship cannot be ruled out.<br>All cases judged by either the reporting medically qualified professional or the Sponsor as having a reasonable suspected causal relationship to the trial medication qualify as adverse reactions.
Serious Adverse Event (SAE)	A serious adverse event is any untoward medical occurrence that:<br>• results in death<br>• is life-threatening<br>• requires inpatient hospitalisation or prolongation of existing hospitalisation<br>• results in persistent or significant disability/incapacity<br>• consists of a congenital anomaly or birth defect.<br>Other 'important medical events' may also be considered serious if they jeopardise the participant or require an intervention to prevent one of the above consequences.<br>NOTE: The term "life-threatening" in the definition of "serious" refers to an event in which the participant was at risk of death at the time of the event; it does not refer to an event which hypothetically might have caused death if it were more severe.
Serious Adverse Reaction (SAR)	An adverse event that is both serious and, in the opinion of the reporting Investigator, believed with reasonable probability to be due to one of the trial treatments, based on the information provided.
Suspected Unexpected Serious Adverse Reaction (SUSAR)	A serious adverse reaction, the nature and severity of which is not consistent with the information about the medicinal product in question set out:<br>• in the case of a product with a marketing authorisation, in the summary of product characteristics (SmPC) for that product<br>• in the case of any other investigational medicinal product, in the investigator's brochure (IB) relating to the trial in question.

NB: to avoid confusion or misunderstanding of the difference between the terms "serious" and "severe", the following note of clarification is provided: "Severe" is often used to describe intensity of a specific event, which may be of relatively minor medical significance. "Seriousness" is the regulatory definition supplied above.

11.2. Causality

The relationship of each adverse event to the trial medication must be determined by a medically qualified individual according to the following definitions:

Unrelated – where an event is not considered to be related to the IMP

Possibly – although a relationship to the IMP cannot be completely ruled out, the nature of the event, the underlying disease, concomitant medication or temporal relationship make other explanations possible.

Probably – the temporal relationship and absence of a more likely explanation suggest the event could be related to the IMP.

Definitely – the known effects of the IMP, its therapeutic class or based on challenge testing suggest that the IMP is the most likely cause.

All AEs (SAEs) labelled possibly, probably or definitely will be considered as related to the IMP.

The final decision relating to causality must be made by a medically qualified Investigator who is a member of the study team.

11.3. Procedures for Recording Adverse Events and Reporting Serious Adverse Events

The safety reporting window for this trial will be from administration of intervention to 6 hours post administration or discharge (whichever is sooner). All trials run by the NPEU Clinical Trials Unit (NPEU CTU) follow the unit's safety reporting Standard Operating Procedure (Safety Reporting in Trials using IMPs). Specific arrangements for this trial are summarised as follows:

Recording adverse events:

Non-serious adverse events will not be routinely recorded as the IMP is a licensed product which is being given at a standard dose. However adverse events which are part of the study outcomes will be recorded in the CRF.

Reporting Serious Adverse Events

All SAEs will be reported immediately, at least within 24 hours; except the following SAEs which are not considered to be causally related to the trial intervention:

- Birth defect/congenital anomaly
- Hypertensive disorder of pregnancy (e.g. pre-eclampsia/eclampsia)
- PPH with onset before the intervention

The SAEs noted above that are not considered due to the trial intervention do not require reporting because these events occurred prior to the trial intervention being administered.

Procedure for immediate reporting of Serious Adverse Events

- Site study staff will report all SAEs except those not considered to causally related to the trial intervention (listed above) to NPEU CTU immediately, at least within 24 hours of the research site becoming aware of the event.
- SAEs can be reported in one of the following ways:
  i. using the Clinical Database OpenClinica©, only staff with access to OpenClinica© may report SAEs in this way, site staff will be required to print off the OpenClinica© SAE form and obtain the information and signature of the Study Clinician carrying out the causality assessment.
  ii. by completing an SAE form which is emailed or faxed to NPEU CTU. Paper copies will be available with the trial documentation to enable anyone to report an SAE.
- Follow-up information should be reported on a new SAE form and this forwarded to the NPEU CTU by fax or email or reported using OpenClinica©.

- The Chief Investigator or safety delegate will review all SAEs and assesses the causality and expectedness of the event in relation to the Reference Safety Information for the Investigational Medicinal Product.
- Review of SAEs will be timely, taking into account the reporting time for a potential SUSAR.
- Site study staff will receive training on the safety reporting process defined in the protocol at their site initiation contact.

11.4. Expectedness

Expectedness will be determined according to the up-to-date Summary of Product Characteristics for co-amoxiclav.

11.5. SUSAR Reporting

All SUSARs will be reported by NPEU CTU to the relevant Competent Authority and to the REC and other parties as applicable. For fatal and life-threatening SUSARS, this will be done no later than 7 calendar days after the Sponsor or delegate is first aware of the reaction. Any additional relevant information will be reported within 8 calendar days of the initial report. All other SUSARs will be reported within 15 calendar days.

Treatment codes will be unblinded for specific participants.

Principal Investigators will be informed of all SUSARs for the relevant IMP for all studies with the same Sponsor, whether or not the event occurred in the current trial.

11.6. Safety Monitoring Committee

The Data Monitoring Committee (see 15.6) will be responsible for safety monitoring. The DMC will conduct a review of all immediately reported SAEs at each meeting and cumulatively to evaluate the risk of the trial continuing and take appropriate action where necessary.

11.7. Development Safety Update Reports

The CI will submit (in addition to the expedited reporting above) DSURs annually throughout the trial, or on request to the Competent Authority (MHRA in the UK), Ethics Committee, Host NHS Trust and Sponsor.

12. STATISTICS

A Statistical Analysis Plan will be produced separately, prior to unblinding of data for the first interim analysis, to be approved by the TSC following review and comments from the DMC. This Statistical Analysis Plan will detail the frequency of the interim analyses for the DMC.

12.1. The Number of Participants

Existing literature suggests a conservative estimate of the background rate of maternal infection following operative delivery of 4% (Liabsuetrakul, Choobun et al. 2004). We have assumed an estimated relative risk reduction of 50% in this rate with antibiotics to 2% in the treatment arm (the single trial relating to operative delivery suggests a greater reduction than this, but this rate of reduction is based on that seen in the more robust antibiotic prophylaxis for caesarean section trials (Smaill and Gyte 2010)). To detect such a difference with 90% statistical power at the two-sided 5% level of significance requires 1,626 per group; with an estimated 5% loss to follow-up the trial would require 1,712 per group, a total of 3,424 women. The planned recruitment curve is shown in Appendix A.

12.2. Description of Statistical Methods

Demographic and clinical data will be summarised with counts and percentages for categorical variables, means (standard deviations) for normally distributed continuous variables and medians (with interquartile or simple ranges) for other continuous variables.

Women will be analysed in the groups to which they were randomly assigned, comparing the outcome of all women allocated to active treatment with all those allocated to placebo, regardless of deviation from the protocol or treatment received (referred to as the Intention to Treat (ITT) population).

For the main analyses, binary outcomes will be reported using unadjusted risk ratios, whilst approximately normally distributed continuous outcomes will be analysed using a t-test and reported using unadjusted mean differences. For excessively skewed continuous outcomes median differences will be presented instead. 95% CIs will be presented for analyses of the primary outcome and 99% CIs for secondary outcomes. Any sensitivity analyses requiring adjustment will be performed using log binomial regression for binary outcomes and linear regression for continuous outcomes.

Loss to follow-up is expected to be a maximum of 5% for short-term outcomes up to six weeks. A pre-specified sensitivity analysis will be undertaken, examining the primary outcome restricted to women who had not received antibiotics in the seven days prior to delivery, in case any masking of a prophylactic effect is occurring by inclusion of pre-treated women.

Since randomisation is performed without minimisation or stratification the primary analysis will not be adjusted for other factors, however, a sensitivity analysis will be conducted including centre as a random effect.

12.3. The Level of Statistical Significance

Two sided statistical testing will be performed throughout. A 5% level of statistical significance will be used for analyses of the primary outcome, and 1% for secondary outcomes.

12.4. Early Trial Cessation

A recommendation may be made by the Data Monitoring Committee to the Trial Steering Committee to stop the trial early following review of interim analysis or evidence from other relevant studies becoming available. Guidelines for the early cessation of the trial will be agreed with the DMC and documented in the DMC Charter.

12.5. Dealing with Missing Data.

Missing data as a result of women being lost to follow-up is expected to be minimal. All reasonable efforts will be taken to minimise loss to follow-up which is expected to be no more than 5%. Women for whom no follow-up primary outcome data are received will be compared to women with data on demographic and clinical characteristics to assess any potential bias due to the impact of the missing data. As there is expected to be a link between outcome and loss to follow-up, imputation techniques will not provide any meaningful information.

12.6. Procedures for Reporting any Deviation(s) from the Original Statistical Plan

All deviations from the original statistical plan will be reported in the final report, as appropriate.

13. DATA MANAGEMENT

13.1. Source Data

Source documents are where data are first recorded, and from which participants' eCRF data are obtained, whether electronic or paper records. These include, but are not limited to, hospital records (from which medical history and previous and concurrent medication may be summarised into the eCRF), clinical and office charts, laboratory and pharmacy records, diaries, microfiches, radiographs and correspondence.

eCRF entries will be considered source data if the eCRF is the site of the original recording (e.g. there is no other written or electronic record of data). All documents will be stored safely in confidential conditions. On all trial-specific

documents, other than the signed consent, the participant will be referred to by the trial participant number/code, not by name.

13.2. Access to Data

Direct access will be granted to authorised representatives from the Sponsor, host institution and the regulatory authorities to permit trial-related monitoring, audits and inspections.

13.3. Data Recording and Record Keeping

All trial data will be entered in to eCRFs. SOPs are in place for the collection and handling of data received at the NPEU CTU. The CI will take overall responsibility for ensuring that each participant's information remains confidential. All paper documents will be stored securely and kept in strict confidence in compliance with the Data Protection Act (1998). Data collected on the eCRFs will be stored in an electronic database in which the participant will be identified only by a trial specific number. The woman's name and any other identifying details will be stored in a separate database linked only by the trial number. After the trial has been completed and the reports published, the data will be archived in a secure physical or electronic location with controlled access.

Storage will be on a restricted area of a file server. The server is in a secure location and access is restricted to a few named individuals. Access to the building in which the NPEU CTU is situated is via an electronic tag and individual rooms are kept locked when unoccupied. Authorisation to access restricted areas of the NPEU network is as described in the NPEU security policies.

Data will be processed on a workstation by authorised staff. The computer workstations access the network via a login name and password (changed regularly). No data are stored on individual workstations. Backing up is done automatically overnight to an offsite storage area. The location of the back-up computer is in a separate department which has electronic tag access. Access to the room in which the back-up machine is located is via a key-pad system.

All essential documents will be retained for at least 5 years after the completion of study-related activities, or for a longer period where so required e.g. genetic studies or national laws, as specified in the NPEU archiving SOP.

14. QUALITY ASSURANCE PROCEDURES

14.1. Risk Assessment

NPEU CTU has performed a risk assessment of the trial prior to commencement that will be reviewed at regular intervals during the course of the trial. This is a trial involving a medicinal product licensed in the UK related to the licensed range of indications, dosage and form; it is proposed that the trial be considered to be of Type A (risk no higher than that of normal clinical practice).

14.2. National Registration Systems

All women recruited into ANODE will be 'flagged' after discharge to confirm status using records held and maintained by The Health and Social Care Information Centre and other central UK NHS bodies.

14.3. Site Initiation and Training

Start-up visits at each participating centre to ensure training in trial procedures will be performed either in person or remotely before recruitment of women is permitted. Regular site visits will be made by the Local Research Midwife (LRM) to ensure adherence to the protocol and to deal with any specific site issues. Study days will be undertaken to ensure that doctors and midwives involved with the study are fully apprised of issues such as informed consent, data collection, follow-up, and changing regulations.

14.4. Data Collection and Processing

All trial data will be collected using bespoke eCRFs. Data will be processed in line with the NPEU CTU Data Management SOPs, using validated data management systems to ensure consistency, viability, and quality of the data. It is then stored in line with the Data Protection Act (1998).

14.5. Central and Site Monitoring

A monitoring plan for the trial, including responsibilities, will be developed in light of any risks identified in the risk assessment, prior to the start of recruitment.

15. TRIAL GOVERNANCE

15.1. Site Research and Development Approval

Individual sites will only commence recruiting participants once they receive approval from NHS Trust Research and Development (R&D) Offices. Applications to R&D offices will be submitted through the NIHR Co-ordinated System for gaining NHS permission.

15.2. Trial Sponsor

The University of Oxford is the nominated sponsor for the trial.

15.3. Co-ordinating Centre

The trial co-ordinating centre will be at the NPEU CTU, University of Oxford where the Trial Co-ordinator will be based. The NPEU CTU will be responsible for all trial programming, randomisation and management, conducting statistical analyses, servicing both the DMC and TSC, and, in collaboration with the CI and the Trial Research Nurse, for the day-to-day running of the trial including recruitment of centres and training of staff.

15.4. Project Management Group

The trial will be supervised on a day-to-day basis by the PMG. This group reports to the TSC which is responsible to the trial sponsor. At each participating centre, a local PI will report to the PMG via the project funded staff based at the NPEU CTU.

The core PMG will consist of the CI and NPEU CTU staff including but not limited to:

- NPEU CTU Director
- Senior Trials Manager
- Trials Programmer
- Trial Coordinator
- Trial Statistician
- Administrator/Data Manager

The core PMG will meet regularly (at least monthly). Every 3–4 months the Clinical Investigators' Group, (CIG) will meet. This will comprise all co-applicants and members of the core PMG.

15.5. Trial Steering Committee

Trial Steering Committee (TSC) will include an independent chair, at least two other independent members, a PPI representative(s), and the Chief investigator, joined by observers from the NPEU CTU. The HTA programme manager will be invited to attend all TSC meetings.

15.6. Data Monitoring Committee

A DMC independent of the applicants and of the TSC will review the progress of the trial at least annually and provide advice on the conduct of the trial to the TSC and (via the TSC) to the HTA programme manager. The DMC will act according to its Charter, which will be agreed at its first meeting.

16. SERIOUS BREACHES

The Medicines for Human Use (Clinical Trials) Regulations contain a requirement for the notification of "serious breaches" to the MHRA within 7 days of the Sponsor becoming aware of the breach.

A serious breach is defined as "A breach of GCP or the trial protocol which is likely to affect to a significant degree –

(a) the safety or physical or mental integrity of the subjects of the trial; or

(b) the scientific value of the trial".

In the event that a serious breach is suspected the Sponsor must be contacted within 1 working day. In collaboration with the CI, the serious breach will be reviewed by the Sponsor and, if appropriate, the Sponsor will report it to the REC committee, Regulatory authority and the NHS host organisation within seven calendar days.

17. ETHICAL AND REGULATORY CONSIDERATIONS

17.1. Declaration of Helsinki

The Investigator will ensure that this trial is conducted in accordance with the principles of the Declaration of Helsinki.

17.2. Guidelines for Good Clinical Practice

The Investigator will ensure that this study is conducted in accordance with relevant regulations and with Good Clinical Practice.

17.3. Approvals

The protocol, informed consent form, participant information sheet and any proposed advertising material will be submitted to an appropriate Research Ethics Committee (REC), regulatory authorities (MHRA in the UK), the funder, and host institution(s) for written approval.

The Investigator will submit and, where necessary, obtain approval from the above parties for all substantial amendments to the original approved documents.

17.4. Reporting

The CI shall submit once a year throughout the clinical trial, or on request, Progress Reports to the REC, host organisation and Sponsor. Six monthly progress reports will be submitted to the funder. In addition, an End of Trial notification and final report will be submitted to the MHRA, the REC, host organisation, funder and Sponsor.

17.5. Participant Confidentiality

The trial staff will ensure that participant anonymity is maintained. All documents will be stored securely and only accessible by trial staff and authorised personnel. The trial will comply with the Data Protection Act, which requires data to be anonymised as soon as it is practical to do so.

17.6. Expenses and Benefits

There are no intended payments or other benefits to participants.

18. FINANCE AND INSURANCE

18.1. Funding

The National Institute for Health Research (NIHR) Health Technology Assessment (HTA) programme is funding the trial.

18.2. Insurance

The University has a specialist insurance policy in place which would operate in the event of any participant suffering harm as a result of their involvement in the research (Newline Underwriting Management Ltd, at Lloyd's of London). NHS indemnity operates in respect of the clinical treatment which is provided.

19. PUBLICATION POLICY

The success of the trial depends on a large number of midwives, obstetricians and anaesthetists. Credit for the study findings will be given to all who have collaborated and participated in the study including all local co-ordinators and collaborators, members of the trial committees, the NPEU CTU, and trial staff. Authorship at the head of the primary results paper will take the form [name], [name]…and [name] on behalf of the ANODE Collaborative Group, where named authors form part of the writing committee. The writing will be the responsibility of the writing committee which it is anticipated will include all of the investigators. Named authors will be listed in the following order: individual responsible for completing the first draft of the paper, lead analyst, all other members of the writing committee in alphabetical order, lead supervising author. All other contributors to the study will be listed at the end of the report, with their contribution to the study identified.

Those responsible for other publications reporting specific aspects of the study, such as detailed microbiological outcomes, may wish to utilise a different authorship model. Decisions about authorship of additional papers will be discussed and agreed by the trial investigators and the TSC.

Women will be sent a summary of trial publications if they wish, which will contain full references. A copy of the journal article will be available on request from the CI.

20. REFERENCES

Acosta, C., S. Bhattacharya, D. Tuffnell, J. Kurinczuk and M. Knight (2012). "Maternal sepsis: a Scottish population-based case–control study." BJOG: An International Journal of Obstetrics & Gynaecology **119**(4): 474-483.

Acosta, C., J. Kurinczuk, D. Lucas, S. Sellers and M. Knight (2013). "Incidence, causes and outcomes of severe maternal sepsis morbidity in the UK." Arch. Dis. Child. Fetal Neonatal Ed. **98(Suppl 1)**: A2.

Acosta, C. D., M. Knight, H. C. Lee, J. J. Kurinczuk, J. B. Gould and A. Lyndon (2013). "The continuum of maternal sepsis severity: incidence and risk factors in a population-based cohort study." PLoS One **8**(7): e67175.

Acosta, C. D., J. J. Kurinczuk, D. N. Lucas, D. J. Tuffnell, S. Sellers, M. Knight and S. United Kingdom Obstetric Surveillance (2014). "Severe maternal sepsis in the UK, 2011-2012: a national case-control study." PLoS Med **11**(7): e1001672.

Bahl, R., B. Strachan and D. Murphy. (2011). "RCOG Green-top Guideline number 26: Operative Vaginal Delivery." Retrieved 23/10/2013, 2013, from http://www.rcog.org.uk/womens-health/clinical-guidance/operative-vaginal-delivery-green-top-26.

Centers for Disease Control and Prevention. (2013). "CDC/NHSN Surveillance Definitions for Specific Types of Infections " Retrieved 15/11/2013, 2013, from http://www.cdc.gov/nhsn/pdfs/pscmanual/17pscnosinfdef_current.pdf.

Chief Medical Officer (2013). Annual Report of the Chief Medical Officer, Volume Two, 2011, Infections and the rise of antimicrobial resistance. London, Department of Health.

Declercq, E., M. Barger, H. J. Cabral, S. R. Evans, M. Kotelchuck, C. Simon, J. Weiss and L. J. Heffner (2007). "Maternal outcomes associated with planned primary cesarean births compared with planned vaginal births." Obstet Gynecol **109**(3): 669-677.

European Centre for Disease Control and Prevention. (2011). "Poor pregnancy outcomes associated with maternal infection with the A(H1N1) 2009 virus during the pandemic – findings from a European cohort study." Retrieved 31/07/2011, 2011, from http://www.ecdc.europa.eu/en/activities/sciadvice/Lists/ECDC%20Reviews/ECDC_DispForm.aspx?List=512ff74f-77d4-4ad8-b6d6-bf0f23083f30&ID=1157&MasterPage=1.

Gulmezoglu, A. M. and L. Duley (1998). "Use of anticonvulsants in eclampsia and pre-eclampsia: survey of obstetricians in the United Kingdom and Republic of Ireland." BMJ **316**(7136): 975-976.

Health and Social Care Information Centre. (2012). "NHS Maternity Statistics - England, 2011-2012." Retrieved 07/09/2013, from http://www.hscic.gov.uk/searchcatalogue?productid=10061&q=maternity+statistics&sort=Relevance&size=10&page=1#top.

Herdman, M., C. Gudex, A. Lloyd, M. Janssen, P. Kind, D. Parkin, G. Bonsel and X. Badia (2011). "Development and preliminary testing of the new five-level version of EQ-5D (EQ-5D-5L)." Qual Life Res **20**(10): 1727-1736.

Hoefman, R. J. and W. Brouwer. (2011). "iMTA Valuation of Informal Care Questionnaire (iVICQ)." Retrieved 17/11/2013, from http://www.bmg.eur.nl/english/imta/publications/questionnaires_manuals/ivicq/.

Johnson, A., R. Thakar and A. H. Sultan (2012). "Obstetric perineal wound infection: is there underreporting?" Br J Nurs **21**(5): S28, S30, S32-25.

Lewis, G. E., R. Cantwell, T. Clutton-Brock, G. Cooper, A. Dawson, J. Drife, D. Garrod, A. Harper, D. Hulbert, S. Lucas, J. McClure, H. Millward-Sadler, J. Neilson, C. Nelson-Piercy, J. Norman, C. O'Herlihy, M. Oates, J. Shakespeare, M. de Swiet, C. Williamson, V. Beale, M. Knight, C. Lennox, A. Miller, D. Parmar, J. Rogers and A. Springett (2011). "Saving Mothers' Lives: Reviewing maternal deaths to make motherhood safer: 2006-2008. The Eighth Report of the Confidential Enquiries into Maternal Deaths in the United Kingdom." BJOG **118 Suppl 1**: 1-203.

Liabsuetrakul, T., T. Choobun, K. Peeyananjarassri and M. Islam (2004). "Antibiotic prophylaxis for operative vaginal delivery." Cochrane Database Syst Rev(3): CD004455.

McCandlish, R., U. Bowler, H. van Asten, G. Berridge, C. Winter, L. Sames, J. Garcia, M. Renfrew and D. Elbourne (1998). "A randomised controlled trial of care of the perineum during second stage of normal labour." Br J Obstet Gynaecol **105**(12): 1262-1272.

McCulloch, P., D. G. Altman, W. B. Campbell, D. R. Flum, P. Glasziou, J. C. Marshall, J. Nicholl, J. K. Aronson, J. S. Barkun, J. M. Blazeby, I. C. Boutron, P. A. Clavien, J. A. Cook, P. L. Ergina, L. S. Feldman, G. J. Maddern, B. C. Reeves, C. M. Seiler, S. M. Strasberg, J. L. Meakins, D. Ashby, N. Black, J. Bunker, M. Burton, M. Campbell, K. Chalkidou, I. Chalmers, M. de Leval, J. Deeks, A. Grant, M. Gray, R. Greenhalgh, M. Jenicek, S. Kehoe, R. Lilford, P. Littlejohns, Y. Loke, R. Madhock, K. McPherson, J. Meakins, P. Rothwell, B. Summerskill, D. Taggart, P. Tekkis, M. Thompson, T. Treasure, U. Trohler and J. Vandenbroucke (2009). "No surgical innovation without evaluation: the IDEAL recommendations." Lancet **374**(9695): 1105-1112.

Morgan, M., R. Hughes and S. Kinsella. (2012). "Green-top Guideline No. 64b: Bacterial Sepsis following Pregnancy." Retrieved 23/10/2013, 2013, from http://www.rcog.org.uk/womens-health/clinical-guidance/sepsis-following-pregnancy-bacterial-green-top-64b.

National Institute for Health and Clinical Excellence. (2007). "Intrapartum Care: NICE Clinical Guideline 55." Retrieved 23/10/2013, 2013, from http://guidance.nice.org.uk/CG55.

National Institute for Health and Clinical Excellence (2011). Caesarean section. NICE Clinical guideline 132. Manchester, National Institute for Health and Clinical Excellence.

Office for National Statistics (2003). Ethnic group statistics: a guide for the collection and classification of ethnicity data. Newport, Office for National Statistics.

Perkins, E., S. Tothill, C. Kettle, D. Bick and K. Ismail (2008). "Women’s views of important outcomes following perineal repair." BJOG **115**(Supplement 1): 152.

Public Health England (Health Protection Agency). (2013). "Protocol for the Surveillance of Surgical Site Infection ", from http://www.hpa.org.uk/webc/HPAwebFile/HPAweb_C/1194947388966.

Smaill, F. M. and G. M. Gyte (2010). "Antibiotic prophylaxis versus no prophylaxis for preventing infection after cesarean section." Cochrane Database Syst Rev(1): CD007482.

Stensballe, L. G., J. Simonsen, S. M. Jensen, K. Bonnelykke and H. Bisgaard (2013). "Use of antibiotics during pregnancy increases the risk of asthma in early childhood." J Pediatr **162**(4): 832-838 e833.

Waterstone, M., S. Bewley and C. Wolfe (2001). "Incidence and predictors of severe obstetric morbidity: case-control study." BMJ **322**(7294): 1089-1093; discussion 1093-1084.

21. APPENDIX A: PLANNED RECRUITMENT

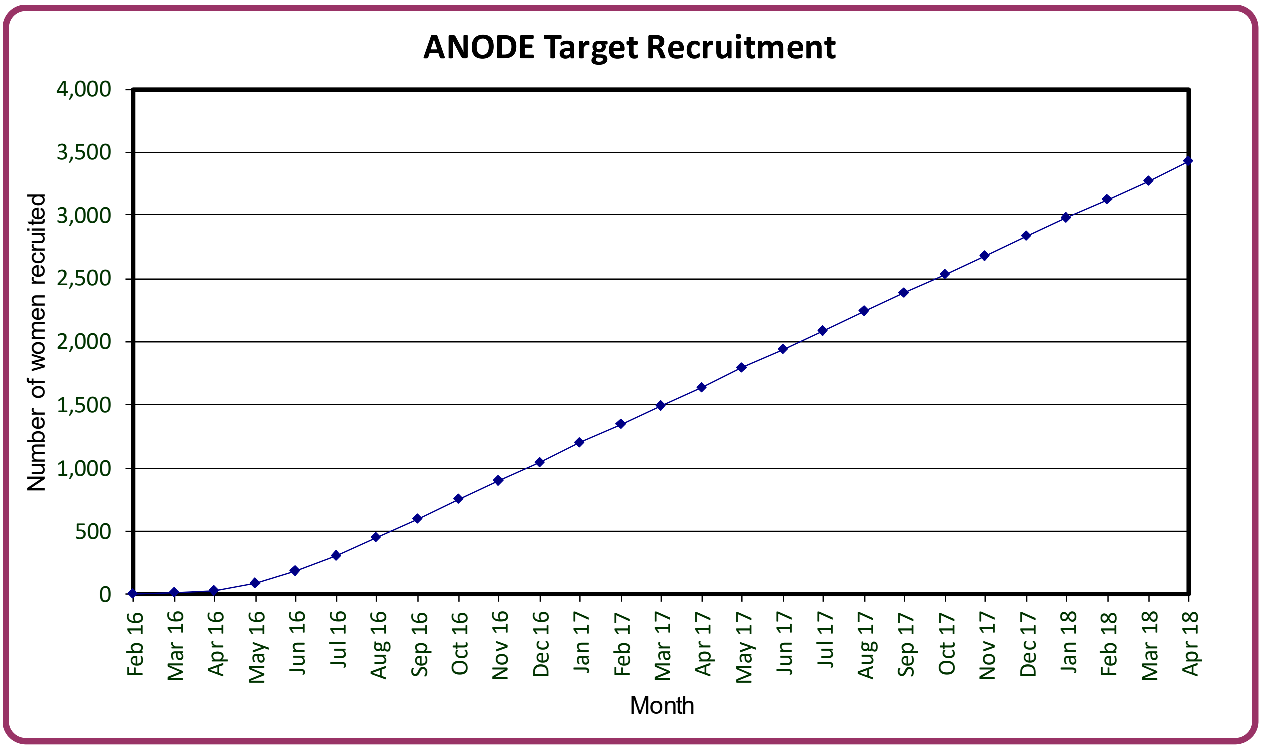

22. APPENDIX B: AMENDMENT HISTORY

<table>
<tr><th>Amendment No.</th><th>Protocol Version No.</th><th>Date issued</th><th>Author(s) of changes</th><th>Details of Changes made</th></tr>
<tr><td>N/A</td><td>Version 2.0</td><td>N/A</td><td>Changes made of behalf of PMG</td><td>Subsequent to Protocol Version 1.0 being approved by REC the MHRA requested several edits to the ANODE Protocol when it was submitted as part of the initial Clinical Trials Authorisation (CTA) application. These edits were made to create Version 2.0 17th October 2015 (see summary of changes below). Version 2.0 17th October 2015 was approved by the MHRA and CTA has been awarded (CTA acceptance letter dated 29th October 2015).</td></tr>
<tr><td colspan="5">The following changes have been made to create Protocol Version 2.0 17th October 2015:

8.3. Exclusion Criteria (page 16)
The exclusion criterion was amended to exclude all participants who have the contraindications listed in the SmPC for Co-amoxiclav as requested by the MHRA.

Reporting Serious Adverse Events (SAEs) and Procedure for immediate reporting of Serious Adverse Events (pages 25-26):
The section was amended in response to a request made by the MHRA on reviewing the initial ANODE CTA application:

'The protocol stating some serious adverse events (SAEs) exempted from immediate reporting to the sponsor is not acceptable. According to Article 16(1) of Directive 2001/20/EC and CT-3, No. 20, the investigator must report all SAEs immediately to the sponsor within 24 hours of awareness, irrespective of causal relationship. Therefore, the exemptions must be removed.'

Edits were made to address this point; the list of SAEs which are not considered to be causally related to the trial intervention, were agreed with Professor Bhattacharya chair of the ANODE Data Monitoring Committee. The SAE reporting procedure was also amended to include the ability to report SAEs via the Clinical Database OpenClinica© on page 26.

Listed below are all edits reviewed by the MHRA prior to the CTA being granted (ANODE Protocol Version 2.0):

1. The Ethics Reference number was been added to page 1.
2. Social care visits were removed from the secondary outcomes; they had been included in error.
3. The spelling of amoxicillin has been made consistent throughout the document.
4. The duration of the study on page 16 was amended to reflect changes made to the recruitment start date (changed from the 1st September 2015 to 1st December 2015).
5. A reference was deleted on page 34 of the Protocol because it was duplicated in error.</td></tr>
<tr><td>1</td><td>Version 3.0</td><td>06/01/16</td><td>Changes made of behalf of PMG</td><td>Substantial amendment 1 was reviewed by both REC and MHRA.</td></tr>
</table>

The following changes have been made to create Protocol Version 3.0 3rd December 2015:

8.3. Exclusion Criteria (page 16)
In the point 'Note that receiving antenatal ~~or postnatal~~ antibiotics e.g. for maternal Group B Streptococcal carriage or prolonged rupture of membranes, is not a reason for exclusion if there is no indication for ongoing antibiotic prescription post-delivery.' the words 'or postnatal' have been removed because this wording was incorrect and contradicts the previous sentence.

Reporting Serious Adverse Events (SAEs) and Procedure for immediate reporting of Serious Adverse Events (pages 25-26):
Wording amended improve consistency and to make it clear that events which commence prior to the administration of the trial Intervention do not require reporting as an SAE.

9.5. Randomisation, blinding and code-breaking (pages 20)
Text edited regarding balance and unpredictability from 'within centre' to 'overall' by Trial Statistician.

Text edited to show that an emergency code-breaking procedure will not be required; as only a single dose of co-amoxiclav will be administered there is no need to code-break if further antibiotics are required. If a woman was to have an anaphylactic reaction she would be treated as if she has been given the active drug.

Other edits to the Protocol in Version 3.0 are listed below:

1. The list of Investigators has been removed from the cover page to make the Protocol clearer and to ensure that the ANODE Trial team at the Clinical Trials Unit are approached with any Protocol queries in the first instance rather than a Co-investigator. The Investigators will be listed on the ANODE website.
2. The confidentiality statement has been removed from page 2 as the Protocol is no longer confidential and is available publicly.

4	Version 4.0	22/04/16	Changes made of behalf of PMG	Changes listed below

The following changes have been made to create Protocol Version 4.0 22nd April 2016:

Section 7.0 Trial Design (page 15)
Edits to this section have been made to specify exactly who will not be blinded to allocation to clarify that this also includes the person responsible for checking the intervention. The people not blinded to intervention will be the person who prepared the trial intervention and the person who checks the intervention prior to administration. Training will be provided to all unblinded staff on the importance of maintaining blinding and unblinded staff will not be involved in the collection of outcomes information.

Other edits to the ANODE Protocol
- Page 1 - ISRCTN added and signature blocks for the Chief Investigator and the Statistician have been removed; they will be documented separately and filed with the Protocol in the Trial Master File.
- Global edit - where Hospital Episode Statistics (HES) is referenced 'or NHS Wales Informatics Service' has been added. HES will be accessed for participants recruited in England; NHS Wales Informatics Service will be accessed for those participants recruited in Wales.
- Pages 15-16 and Appendix A - duration of study edited to reflect the change in start date agreed with the HTA.
- Page 19 - edited to clarify that the original signed consent forms will be sent the coordinating centre and a copy retained at site.
- Page 20 - section 9.5 edited to clarify the roles of the Senior Trials Statistician and the Senior Trial Programmer with regard to their responsibilities regarding the randomisation schedule generation.

13	Version 5.0	30/11/17	Changes made of behalf of PMG	Changes listed below

The following changes have been made to create Protocol Version 5.0 30th November 2017:

Amendment to the definition of the primary outcome (pages 7, 9 (in flowchart), 14 and 20)
Primary outcome refined to the amended text below:

- A new prescription of antibiotics for presumed perineal wound-related infection, endometritis or uterine infection, urinary tract infection with systemic features or other systemic infection
- Confirmed systemic infection on culture
- Endometritis as defined by the US Centers for Disease Control and Prevention (Centers for Disease Control and Prevention 2013)

Trial timeline updated to reflected changes following an extension to the duration of the trial (page 16 and page 39)

12.2 Description of Statistical Methods (page 27)
The statistics section of the protocol has been updated to make it consistent with the current strategy detailed in the statistical analysis plan as requested by the ANODE Data Monitoring Committee in a meeting held on the the 27th November 2017.

ANODE

A randomised controlled trial of prophylactic ANtibiotics to investigate the prevention of infection following Operative vaginal DElivery

Statistical Analysis Plan
Version 1.0
Date: 01/11/2018

Authors: *Dr Christopher Partlett, Medical Statistician, NPEU CTU*
Virginia Chiocchia, Medical Statistician, NPEU CTU
Reviewers: *Dr Louise Linsell, Lead Medical Statistician, NPEU CTU*
Professor Marian Knight, Chief Investigator, NPEU
Associate Professor Ed Juszczak, Director, NPEU CTU

Protocol version: Version 5.0

Background: ANODE is a multi-centre placebo-controlled randomised controlled trial (RCT) to assess whether the administration of a prophylactic antibiotic can reduce the incidence of confirmed or suspected maternal infection in the first six weeks after operative vaginal delivery.

TABLE OF CONTENTS

1 Introduction 42
2 Trial Personnel 42
3 Background Information 42
3.1 Rationale 42
3.2 Aims of the trial 43
3.3 Trial design 43
3.4 Eligibility 43
3.5 Interventions 43
3.6 Definition of primary and secondary outcomes 44
3.7 Hypothesis framework 44
3.8 Sample size & power 45
3.9 Treatment allocation 45
3.10 Data collection schedule 45
3.11 Data entry, cleaning and validation 46
3.12 Interim analyses and stopping rules 46
3.13 Trial reporting 47
4 Protocol non-compliances 47
4.1 Major 47
4.2 Minor 47
5 Adherence to the Intervention 48
6 Patient Groups for Analysis 48
7 Descriptive Analyses 48
7.1 Representativeness of trial population and participant throughput 48
7.2 Baseline comparability of randomised groups 49
7.3 Losses to follow-up 49
7.4 Adherence to intervention 49
8 Comparative Analyses 50
8.1 Evaluation/definition of outcomes 50
8.2 Primary analysis 50
8.3 Secondary analyses 50
8.4 Pre-specified subgroup analyses 51
8.5 Sensitivity analyses 51
8.6 Significance levels and adjustment of p-values for multiplicity 51
8.7 Missing data 51
8.8 Statistical Software Employed 51
9 Safety data analysis 51
10 Additional Exploratory Analysis 52
11 Deviation from Analysis Described in Protocol 52
12 References 52
12.1 Trial documents 52

12.2 Other references 52
13 Approval 53
14 Document history 54

1 INTRODUCTION

This document details the proposed presentation and analyses for reporting the main analysis results from the National Institute for Health Research (NIHR) Health Technology Assessment (HTA) programme funded multicentre randomised controlled trial ANODE.

The results reported in this paper will follow the strategy set out here. Subsequent analyses of a more exploratory nature will not be bound by this strategy, though they are expected to follow the broad principles laid down here. The principles are not intended to curtail exploratory analysis, nor to prohibit accepted practices, but they are intended to establish the rules that will be followed, as closely as possible, when analysing and reporting the trial.

The analysis plan will be available on request when the principal papers are submitted for publication in a journal. Suggestions for subsequent analyses by journal editors or referees, will be considered carefully, and carried out as far as possible in line with the principles of this analysis plan.

Any deviations from the statistical analysis plan will be described and justified in the final report of the trial. The analysis should be carried out by an identified, appropriately qualified and experienced statistician, who should ensure the integrity of the data during their processing. Examples of such procedures include quality control and evaluation procedures.

2 TRIAL PERSONNEL

Trial Statisticians
Christopher Partlett, Medical Statistician, NPEU CTU (until June 2018)
christopher.partlett@npeu.ox.ac.uk

Virginia Chiocchia, Medical Statistician, NPEU CTU (from June 2018)
virginia.chiocchia@npeu.ox.ac.uk

Louise Linsell, Lead Medical Statistician, NPEU CTU
louise.linsell@npeu.ox.ac.uk

Chief Investigator
Marian Knight, Chief Investigator, NPEU

CTU Director
Ed Juszczak, Director, NPEU CTU

Trial management
Shan Gray, ANODE Trial Co-ordinator, NPEU CTU
Nelly Owino, ANODE Trial Data Co-ordinator, NPEU CTU

Trial Programmers
Andy King, Head of Trials Programming, NPEU CTU
David Murray, Senior Trials Programmer, NPEU CTU

3 BACKGROUND INFORMATION

3.1 Rationale

Sepsis is now the most important cause of direct maternal death in the UK [1]. Studies conducted both in the UK and US, have documented an additional risk associated with operative vaginal delivery [2-4], and particularly in relation to Group A streptococcal infection, the leading and most severe cause of maternal infection [1, 4]. A Cochrane review, updated in 2012, has identified only one small previous trial of prophylactic antibiotics following operative vaginal delivery, including a total of 393 women, with a relative risk of 0.07 (95%

confidence interval 0.00 to 1.21) for postpartum infection [5], and given the small study size and extreme result, recommends that further robust evidence is needed.

Thirteen percent of women in the UK undergo forceps or ventouse deliveries [6], an estimated 104,000 women annually. The conservatively estimated incidence of maternal infection following operative vaginal delivery is 4%, based on the one previous trial [5], resulting in an estimated 4,160 women potentially having an infection after instrumental delivery. Of these women, around 200 will be diagnosed with severe sepsis [2], and up to four may die from their infection [1, 4]. There is therefore considerable scope for direct patient benefit from an effective preventive strategy.

3.2 Aims of the trial

The primary aim is to compare the incidence of confirmed or suspected maternal infection in the first six weeks after operative vaginal delivery amongst women who have been randomised to receive a prophylactic antibiotic versus those who received a placebo.

The secondary aims of the trial concern the effect of the intervention on the following maternal outcomes: systemic sepsis, perineal wound infection, perineal pain, use of pain relief, need for additional perineal care, wound breakdown, dyspareunia, breast feeding, ability to sit comfortably to feed the baby, hospital bed stay, GP/home visits, outpatient visits, hospital re-admission, maternal general health, and antibiotic side effects.

3.3 Trial design

ANODE is a blinded, multicentre, randomised, placebo-controlled trial comparing whether a single dose of prophylactic antibiotic following operative vaginal delivery is clinically effective for preventing confirmed or suspected maternal infection in women who have undergone forceps or ventouse delivery at 36^{+0} weeks or greater gestation.

It is anticipated the trial will last for approximately 38 months, recruiting 3,424 women from at least 21 obstetric units within the UK over 24 months.

3.4 Eligibility

<u>Participants</u>
Women who have undergone operative vaginal delivery at 36^{+0} weeks or greater gestation, with no indication for ongoing prescription of antibiotics in the postpartum period and no contra-indications to prophylactic co-amoxiclav.

<u>Inclusion Criteria</u>

- Women aged 16 years or above, willing and able to give informed consent.
- Women who have had an operative vaginal delivery at 36^{+0} weeks or greater gestation.

<u>Exclusion Criteria</u>

- Clinical indication for ongoing antibiotic administration post-delivery, e.g. due to confirmed antenatal infection, 3rd or 4th degree tears. Note that receiving antenatal antibiotics, e.g. for maternal Group B Streptococcal carriage or prolonged rupture of membranes, is not a reason for exclusion if there is no indication for ongoing antibiotic prescription post-delivery.
- Known allergy to penicillin or to any of the components of co-amoxiclav, as documented in hospital notes.
- History of anaphylaxis (a severe hypersensitivity reaction) to another β-lactam agent (e.g. cephalosporin, carbapenem or monobactam), as documented in hospital notes.

3.5 Interventions

<u>Treatment</u>

A single dose of intravenous co-amoxiclav (1g amoxicillin/200mg clavulanic acid). Co-amoxiclav 1,000mg/200mg powder for solution for injection is supplied as bottles of sterile powder for making up as an injection reconstituted with sterile water for injection, also supplied.

Control
A single dose of intravenous sterile saline. Placebo (0.9% saline) will be supplied as 20ml single use vials of clear liquid. Reconstitution is not required.

3.6 Definition of primary and secondary outcomes

Primary outcome
Confirmed or suspected maternal infection within 6 weeks of delivery, as defined by one of:

- Confirmed systemic infection on culture
- Endometritis as defined by the US Centers for Disease Control and Prevention [7]
- A new prescription of antibiotics for presumed perineal wound-related infection, endometritis or uterine infection, urinary tract infection with systemic features or other systemic infection

An episode of endometritis requires meeting at least one of the following criteria:

1. Organisms are cultured from fluid (including amniotic fluid) or tissue from endometrium obtained during an invasive procedure or biopsy
2. Woman exhibits at least two of the following signs or symptoms: fever (>38°C), abdominal pain, uterine tenderness, or purulent drainage from uterus with no other recognised cause

Secondary outcomes
The following additional maternal outcomes (at 6 weeks):

- Systemic sepsis
- Perineal wound infection
- Perineal pain
- Use of pain relief for perineal pain
- Need for additional perineal care
- Wound breakdown
- Dyspareunia
- Breast feeding
- Ability to sit comfortably to feed the baby
- Hospital bed stay
- GP or home visits in relation to perineum
- Outpatient visits in relation to perineum
- Hospital readmission for maternal reason
- Maternal general health (assessed using EQ-5D-5L)
- Common and serious antibiotic side effects from Summary of Product Characteristics (SmPC), including:
    - Anaphylaxis
    - Diarrhoea
    - Nausea
    - Vomiting
    - Skin rash
    - Pruritus
    - Other

In addition, hospital admissions and diagnoses will be obtained at one year post-delivery, although the analysis of these data is not covered by this analysis plan.

3.7 Hypothesis framework

ANODE is a superiority trial, comparing co-amoxiclav with placebo. Analysis of the trial will entail calculation of treatment effect measures and confidence intervals to assess the difference between the two arms.

3.8 Sample size & power

The primary comparison will be the difference in the proportion of women with confirmed or suspected maternal infection within 6 weeks of delivery.

A conservative estimate of the background rate of maternal infection following operative delivery was predicted to be 4% [5]. Assuming an estimated relative risk reduction of 50% in the treatment arm, to detect such a difference with 90% statistical power at the two-sided 5% level of significance requires 1,626 per group. With an estimated 5% loss to follow-up, the trial would require 1,712 per group; a total of 3,424 women.

3.9 Treatment allocation

The allocation ratio of intervention (co-amoxiclav) to control (placebo) arms will be 1:1. A randomisation list will be generated using permuted blocks of variable size to ensure balance and unpredictability overall. Centres will be supplied with sealed sequentially numbered indistinguishable packs containing active drug or placebo (saline solution), as designated. Women will be randomised by the allocation of the next sequentially numbered pack once consent and eligibility are established.

3.10 Data collection schedule

Clinical information will be collected using the following case report forms (CRFs):

- Eligibility Confirmation Form
- Randomisation and Intervention Form
- Woman Outcomes Form
- Infection Form
- 6 Week Telephone Follow-up Script
- 6 Week Follow-up Questionnaire
- Health Records Form
- Blinded Endpoint Review Committee (BERC) Form
- Withdrawal Form
- Serious Adverse Event (SAE) Form

The schedule of trial assessments are shown below. Outcome information will be collected by a single telephone interview and questionnaire, with clinical data collection from medical records or the hospital laboratory if necessary, at six weeks post-delivery.

There will be no further follow-up, but participants will be asked for permission to link their records to Hospital Episode Statistics or NHS Wales Informatics Service in order to assess outcomes at one year.

For women with missing primary outcome data, the answers to the 6 Week Telephone Follow-up Script will be sourced from hospital and/or GP records and entered into the Health Records Form. Where the primary outcome is incomplete but a 6 week questionnaire is available, a blinded endpoint review committee (BERC) will attempt to make a final determination on whether a woman has or has not met the primary outcome. The decision will be recorded on the BERC Form.

Procedure	Eligibility screening	Trial Entry and drug administration (day 1)	Up to 6 hours after trial drug was administered	6 weeks post-delivery
Demography		✓		
Confirmation of Eligibility	✓			
Consent		✓		
Randomisation		✓		
Co-amoxiclav/ Placebo Dosing[1]		✓		
SAEs		✓	✓	
Concomitant Medication[2]		✓	✓	
6 week telephone interview				✓
6 week Questionnaire				✓

[1]Initial trial drug administrations to be given as soon as possible after randomisation.
[2]Concomitant medications to be recorded only in relation to SAEs. In the event of an SAE all concomitant medication, from admission to labour ward to time of event, must be recorded on the SAE form.

3.11 Data entry, cleaning and validation

All trial data are collected and entered using study-specific eCRFs, with the exception of the 6 week questionnaire. Data will be processed in line with the NPEU CTU Data Management SOPs, using validated data management systems to ensure consistency, viability, and quality of the data.

3.12 Interim analyses and stopping rules

A Data Monitoring Committee (DMC), independent of the trial organisers, will review the trial's progress. Interim analyses will be supplied, in strict confidence, to the DMC, as frequently as its Chair requests. The terms of reference for the DMC were agreed at their first meeting, and a DMC Charter was completed and signed by all members. Meetings of the committee will be arranged annually, or more often as appropriate.

The trial statistician will produce (or oversee the production of) closed reports for the DMC and will participate in DMC meetings, guide the DMC through reports, and will also take meeting minutes. A template report/dummy tables will be agreed.

The DMC will receive and review the progress and accruing data of the trial and provide advice on the conduct of the trial to the Trial Steering Committee (TSC). The trial statistician will produce the closed report blinded to allocation (using allocation codes) and it will be at the discretion of the DMC whether or not to reveal the codes and thereby unblind its members and/or the trial statistician.

Unless modification or cessation of the trial is recommended by the DMC, the TSC, investigators, collaborators and administrative staff (except those who supply the confidential information) will remain ignorant of the results of the interim analysis. Collaborators and all others associated with the study may write to the DMC via NPEU CTU, to draw attention to any concern they may have about the possibility of harm arising from the treatment under study. See the DMC Charter for more information.

In the light of interim data and other evidence from relevant studies, the DMC will inform the TSC if, in its view, there is proof beyond reasonable doubt that the data indicate that the trial should be terminated. A decision

to inform the TSC of such a finding will in part be based on statistical considerations. Appropriate proof beyond reasonable doubt cannot be specified precisely. A difference of at least 3 standard errors in the interim analysis of a major endpoint may be needed to justify halting or modifying the study prematurely.

3.13 Trial reporting

The trial will be reported according to the principles of the CONSORT statement. The final analysis will be conducted for all outcomes collectively, at the end of the trial.

4 PROTOCOL NON-COMPLIANCES

All protocol non-compliances will be listed in the final report. Non-compliances are defined below.

4.1 Major

The following will be defined as major protocol non-compliances:

Women without confirmed consent

- Women without a record of verbal or confirmed consent
- Women with a record of verbal consent, but no record of confirmed consent

Participants randomised in error

- Women with a known allergy to penicillin or to any of the components of co-amoxiclav
- Women with a history of anaphylaxis

Evidence of fraud or misconduct

- Data considered fraudulent

4.2 Minor

The following will be defined as minor protocol non-compliances:

Participants randomised in error
These include

- Women who were randomised, but did not go on to have operative vaginal delivery
- Women below the age of 16 at randomisation
- Women with an ongoing antibiotic administration post-delivery

Participants who do not receive allocated intervention
These include

- Women who were allocated to the co-amoxiclav arm, but did not receive co-amoxiclav (i.e. received placebo or no treatment) within six hours of delivery
- Women who were allocated to the placebo arm, but did not receive the placebo (i.e. received co-amoxiclav or no treatment) within six hours of delivery

Follow-up completed outside the set time window

- Telephone or follow-up questionnaires completed before 6 weeks and after 10 weeks post-delivery. This will be determined from the woman's delivery date (recorded on the Confirmation of Eligibility form) and the date the interview or questionnaire was completed. For the postal questionnaire the date of completion is recorded on the form. For the telephone script and online questionnaire the date and time that data entry is started and completed is recorded. For these forms the date at which data is first entered will be used except for cases where data entry for the telephone script started before 6 weeks post-delivery. In this case the date that data entry was completed will be used.

5 ADHERENCE TO THE INTERVENTION

Adherence to the intervention is recorded at trial entry form on the randomisation and entry form. The date and time of the administration of the intervention is also recorded. If women did not receive the allocated intervention within 6 hours of delivery then they are considered to not have adhered to the intervention.

6 PATIENT GROUPS FOR ANALYSIS

Women will be analysed in the groups into which they were randomly allocated, comparing the outcomes of all women allocated to the co-amoxiclav group with those allocated to placebo, regardless of deviation from the protocol or treatment received (referred to as the Intention to Treat (ITT) population).

Post-randomisation exclusions
The following will be excluded from the analysis population post-randomisation:

- Participants for whom full consent was not obtained.
- Participants for whom an entire record of fraudulent data was detected (should fraudulent data be detected, consideration will be given to excluding all data for the site where such data were found).

The numbers (with percentages of the randomised population) of post-randomisation exclusions will be reported by trial arm, and reasons summarised, in the CONSORT flow diagram.

Descriptive analysis population
Baseline demographic and clinical characteristics will be reported for all participants randomised for whom we have data available, excluding post-randomisation exclusions and women who have withdrawn consent to use their data.

Comparative analysis population
All women randomised, excluding post-randomisation exclusions and women who have withdrawn consent to use their data.

Safety population
All women who received co-amoxiclav or placebo.

Interim analysis population
Excluding post-randomisation exclusions and women who have withdrawn consent to use their data:

- Baseline and safety data will be reported for all trial participants with available data.
- Outcomes will be restricted to women with 6 week follow-up data available (i.e. a completed 6 week questionnaire or telephone interview).

For outcomes with multiple derivations these will be assumed negative for the interim analysis unless they can be positively derived by at least one of the derivations. This will be the case even if some data relating to that outcome is missing. Only if the extent of missing data is such that every derivation is indeterminate will the outcome be considered as 'missing'.

7 DESCRIPTIVE ANALYSES

7.1 Representativeness of trial population and participant throughput

The flow of participants through each stage of the trial will be summarised using a CONSORT flow chart. We will report the numbers of participants:

- Total randomised
- Allocated to each intervention
- Post-randomisation exclusions
- Withdrawn
- Randomised in error

- Did not receive allocation
- Followed up at six weeks
  - Interviewed by telephone
  - Completed postal or online questionnaire
- Included at baseline
- Included in the analysis
  - Complete primary outcome data

7.2 Baseline comparability of randomised groups

Participants in the original two randomised groups will be described separately with respect to their demographic and clinical characteristics at trial entry.

Numbers (with percentages) for binary and categorical variables and means (and standard deviations), or medians (with interquartile range, and minimum and maximum values if appropriate) if the data are skewed, for continuous variables will be presented. There will be no tests of statistical significance performed nor confidence intervals calculated for differences between randomised groups on any baseline variable.

This will include the following maternal characteristics

- Maternal demographic and pregnancy characteristics at trial entry
  - Age
  - Gestational age at entry (in completed weeks)
  - Ethnic group (categories are listed in Table 1 of Dummy Tables)
  - BMI (at booking)
- Previous obstetric and medical history
  - Multiple pregnancy
  - Previous pregnancy <22 weeks' gestation
  - Previous pregnancy ≥22 weeks' gestation
  - Previous caesarean section
  - Previous episiotomy
  - Previous tear
- Surgical characteristics of current pregnancy
  - Rupture of membranes before delivery
  - Labour induction
  - Actual mode of delivery
  - Failed instrumental delivery
  - Reason for instrumental delivery
  - Episiotomy in current delivery
  - Tear in current delivery
  - Wound sutured
  - Location of suturing
- Centre

7.3 Losses to follow-up

Baseline information collected on women with incomplete primary outcome data (and subsequently are not included in the analysis of the primary outcome) will be presented alongside those with complete primary outcome data, overall and within treatment group.

Any deaths will be reported separately.

7.4 Adherence to intervention

Adherence to the intervention will be reported for all women, excluding post-randomisation exclusions and women who have withdrawn consent to use their data.

In addition to the timing of the administration the following adherence will be reported

- The frequency that the same person who prescribed antibiotics was responsible for preparing or checking the trial intervention
- The frequency that the telephone interview is administered by the same person who administered the intervention

Details of how these checks are calculated are described in the data derivation spreadsheet.

8 COMPARATIVE ANALYSES

For the primary analysis, women will be analysed in the groups into which they were randomly allocated, comparing the outcomes of all women allocated to trial treatment with those allocated to the control group, regardless of allocation received (referred to as the Intention to Treat (ITT) population).

8.1 Evaluation/definition of outcomes

Derivation of variables is recorded in the data derivation spreadsheet.

Where the primary outcome is incomplete but a 6 week follow questionnaire is available, data in the 6 week questionnaire will be used to derive the primary outcome:

- If a woman has stated in their questionnaire that she has not seen a healthcare worker, has not had any outpatient visits, has not been re-admitted anywhere, and no free text is recorded, this is sufficient to rule out the primary outcome without the need for a BERC.
- Otherwise, a BERC will determine whether a woman has or has not met the primary outcome, where possible.

8.2 Primary analysis

The proportion of women with confirmed or suspected maternal infection within 6 weeks of delivery will be reported in each arm and summarised by a risk ratio, with a 95% confidence interval (CI). A breakdown of the proportion of women with a positive culture, endometritis, and a new prescription of antibiotics will be presented; women who experience more than one of these events will be reported in each.

Randomisation was performed without minimisation or stratification and so the analyses will not be adjusted for other factors. However, as centres were provided with batches of sequential packs, a sensitivity analysis will be conducted, including centre as a random effect.

8.3 Secondary analyses

All secondary binary outcomes will be summarised using risk ratios, with 99% confidence intervals.

Maternal general health, as assessed using EQ-5D-5L, will be summarised in each arm using the overall index value. This will be summarised using a median and interquartile range (IQR) and compared using the difference in medians (and 99% confidence interval) and the Wilcoxon rank-sum test. The number and proportion of women with problems will also be summarised by arm for each domain; however, a statistical comparison will only be performed on the overall index value.

For hospital bed stay, calculated as the total number of nights spent in hospital in the first instance plus all subsequent visits, the median and interquartile range (IQR) will be reported in each arm and compared using the difference in medians (and 99% confidence interval) and the Wilcoxon rank-sum test.

In case of duplicate questionnaires, e.g. when both a postal and an online questionnaire, or two postal questionnaires are available, the following rules will apply.

- If the dates of completion differs on the two questionnaires:
    1. The questionnaire completed earlier will be used, unless the date of completion is before 6 weeks post-discharge; in this case, the later questionnaire should be used.
    2. If there is missing data in the earlier questionnaire, non-missing data from the later questionnaire should be used.

- If the date of completion is the same in the two questionnaires, the most severe answers and/or non-missing data will be combined on the postal questionnaire which should be used for analysis.

8.4 Pre-specified subgroup analyses

There are no pre-specified subgroup analyses.

8.5 Sensitivity analyses

A pre-specified sensitivity analysis will be undertaken, examining the primary outcome restricted to women who had not received antibiotics in the seven days prior to delivery, in case any masking of a prophylactic effect is occurring by inclusion of pre-treated women.

A repeat analysis of the primary outcome will be undertaken, excluding women prescribed antibiotics (other than the trial intervention) within 24 hours of delivery (as discussed at joint TSC/DMC meeting – 9/7/15). The date and time the intervention is administered and date and time of delivery are recorded on the entry form.

A sensitivity analysis, including centre as a random effect, will be carried out (as discussed at DMC meeting – 22/6/17). Also, for the primary outcome, heterogeneity across centres will be investigated using a forest plot.

A repeat analysis of the primary outcome will be undertaken restricted to women whose primary outcome was obtained from a telephone script and/or a BERC form based on a questionnaire completed between 6 and 10 weeks after delivery. Women whose primary outcome was obtained from health records, as described in section 3.10, will be included in this analysis.

8.6 Significance levels and adjustment of p-values for multiplicity

95% confidence intervals will be presented for analyses of the primary outcome. To allow for multiple secondary outcomes, more stringent 99% confidence intervals will be presented.

8.7 Missing data

A number of binary outcomes (YES/NO) have several possible derivations, using data from different forms collected at different time points. For the final analysis, the following rules will be applied to combine the outcomes:

- If at least one derivation is a YES (is indicative of the outcome) this will be treated as a YES.
- On the other hand, all derivations must be NO (rule out the outcome) for the outcome to be treated as a NO.
- All other responses will be treated as missing.

Any women with discrepant derivations may be subject to blinded endpoint review.

A number of outcomes can be derived using the infection form, which is triggered by specific questions on the woman outcomes form (at discharge) or the telephone interview (at 6 weeks). For any derivations involving the infection form these will be treated as NO if there is no indication that an infection form should have been completed from a trigger question AND there are no infection forms present (or a form has been completed but it does not indicate an infection).

8.8 Statistical Software Employed

Analyses will be completed in Stata/SE version 15.1 for Windows.

9 SAFETY DATA ANALYSIS

Serious adverse events will be summarised by trial arm, with numbers of each severity (mild, moderate or severe); causality (unrelated, possibly, probably, definitely); and action taken (to be categorised when data is available).

Events will also be listed, reporting allocation, severity, relatedness to study drug, outcome, and details.

Common and serious antibiotic side effects from Summary of Product Characteristics (SmPC) will be summarised for both arms.

10 ADDITIONAL EXPLORATORY ANALYSIS

Any analyses not specified in the analysis protocol will be exploratory in nature and a 2-sided significance level of 0.01 will be used with 99% confidence intervals.

11 DEVIATION FROM ANALYSIS DESCRIBED IN PROTOCOL

None yet.

12 REFERENCES

12.1 Trial documents

ANODE Dummy Tables
ANODE Data Derivation
ANODE Trial Protocol version 5.0 01Dec17
ANODE DMC Charter Version 1.1 23Feb18
ST105 v2.0 Statistical Analysis Plan

12.2 Other references

1. Lewis, G.E., et al., *Saving Mothers' Lives: Reviewing maternal deaths to make motherhood safer: 2006-2008. The Eighth Report of the Confidential Enquiries into Maternal Deaths in the United Kingdom.* BJOG, 2011. **118 Suppl 1**: p. 1-203.
2. Acosta, C., et al., *Maternal sepsis: a Scottish population-based case–control study.* BJOG: An International Journal of Obstetrics & Gynaecology, 2012. **119**(4): p. 474-483.
3. Acosta, C.D., et al., *The continuum of maternal sepsis severity: incidence and risk factors in a population-based cohort study.* PLoS One, 2013. **8**(7): p. e67175.
4. Acosta, C.D., et al., *Severe maternal sepsis in the UK, 2011-2012: a national case-control study.* PLoS Med, 2014. **11**(7): p. e1001672.
5. Liabsuetrakul, T., et al., *Antibiotic prophylaxis for operative vaginal delivery.* Cochrane Database Syst Rev, 2004(3): p. CD004455.
6. Health and Social Care Information Centre. *NHS Maternity Statistics - England, 2011-2012.* 2012 07/09/2013]; Available from: http://www.hscic.gov.uk/searchcatalogue?productid=10061&q=maternity+statistics&sort=Relevance&size=10&page=1#top.
7. Centers for Disease Control and Prevention. *CDC/NHSN Surveillance Definitions for Specific Types of Infections* 2013 [cited 2013 15/11/2013]; Available from: http://www.cdc.gov/nhsn/pdfs/pscmanual/17pscnosinfdef_current.pdf.

13 APPROVAL

<table>
<tr><td rowspan="2">Senior Trial Statistician</td><td colspan="2">Name:</td></tr>
<tr><td>Signature</td><td>Date</td></tr>
<tr><td rowspan="2">Chief Investigator</td><td colspan="2">Name:</td></tr>
<tr><td>Signature</td><td>Date</td></tr>
<tr><td rowspan="2">Chair of Trial Steering Committee</td><td colspan="2">Name:</td></tr>
<tr><td>Signature</td><td>Date</td></tr>
</table>

14 DOCUMENT HISTORY

Version	Date	Edited by	Comments/Justification	Timing in relation to interim analysis/unblinding
0.1	05/12/2016	CP	Preliminary draft by CP	Prior to both
0.2	15/12/2016	CP	Updated draft following comments from LL	Prior to both
0.3	03/01/2017	CP	Updated following comments from MK	Prior to both
0.4	06/01/2017	CP	Updated following further comments from LL.	Prior to both
0.5	20/01/2017	CP	Updated following further comments from MG	Prior to both
0.6	25/01/2017	CP	Updated following comments from LL	Prior to both
0.7	30/01/2017	CP	Updated following further comments from LL	Prior to both
0.8	15/02/2017	CP	Updated following DMC review	After first interim analysis, prior to unblinding.
0.9	15/02/2017	CP	Updated following comments from LL	After first interim analysis, prior to unblinding.
0.10	15/03/2017	CP	Updated following comments from ORA and EJ	After first interim analysis, prior to unblinding.
0.11	15/08/2017	CP	Updated following comments from the TSC. Data derivation section removed and incorporated into data derivation spreadsheet: (v0.7) 12-04-2017.<br><br>Additional updates made following ANODE DMC on 22-06-17<br><br>Added details regarding sensitivity analysis for primary outcome on 15-08-17	After first interim analysis, prior to unblinding.
0.12	28/11/2017	CP	Made minor changes and added additional comments following DMC on 27-11-17	After first interim analysis, prior to unblinding.
0.13	08/02/2018	CP	Changes made to description of primary outcome following protocol amendment.	After first interim analysis, prior to unblinding.
0.14	27/02/2018	CP	Added a sensitivity analyses (on the recommendation of the DMC – 15/02/18) restricted to primary outcome derived from the telephone script (carried out within 10 weeks)	After second interim analysis, prior to unblinding.
0.15	19/06/2018	CP	Added details of the role of BERC review in the derivation of the primary outcome to section 8.1 (Evaluation/definition of outcomes)	After second interim analysis, prior to unblinding.
0.16	23/10/2018	VC	Added change to trial statistician, change to time window for completion of follow-up, removal of non-consenting women from minor non-compliances, removal of exclusion of questionnaires date	After second interim analysis, prior to unblinding.

			>12 weeks post-delivery from primary analysis.<br>Added decision for outcome derivation in case of duplicates questionnaire in section 8.3.	
0.17	29/10/2018	VC	Changes added following review by LL and MK.<br>Changes to decisions for outcome derivation in case of duplicate questionnaires and to sensitivity analysis following review meeting with MK, NO on 29-10-18	After second interim analysis, prior to unblinding.
0.18	30/10/2018	VC	Updated with minor changes and details following review by LL and MK.<br>Added VC as author on front page.	After second interim analysis, prior to unblinding.
0.19	31/10/2018	VC	Section 3.10 updated with addition of health records and BERC form after discussion with LL and MK.	After second interim analysis, prior to unblinding.
1.0	02/11/2018	LL	Email confirmation from Marian Knight (CI) and Fiona Dennison (TSC Chair) to sign off version 0.19 with no further changes.	After second interim analysis, prior to unblinding.

Table S1: Source of unit costs for different health care resources

Health care resource use item	Unit cost	Sources (notes)
Antibiotics		
Single dose of amoxicillin/clavulanic acid assigned to intervention	2·3	British National Formulary 2017 (average of three brands with NHS indicative prices for 1000mg amoxicillin/200mg clavulanic acid powder for solution for injection vials)
Course of amoxicillin/clavulanic acid assigned to prescriptions following delivery	4·8	British National Formulary 2017 (average of fourteen brands with NHS indicative prices for oral suspension and tablet courses of amoxicillin/clavulanic acid)
Health care professionals		
General practitioner (GP)	37·0	Unit Costs of Health and Social Care 2017
Nurse/midwife at GP practice	10·9	Unit Costs of Health and Social Care 2017 (duration of visit extracted from Unit Costs of Health and Social Care 2015)
Nurse/midwife in home	68·0	NHS reference cost 2017-18 (code N01P)
Health visitor/district nurse	38·0	NHS reference cost 2017-18 (code N02AF)
Outpatient visits		
Outpatient visit doctor	138·2	NHS reference cost 2017-18 (weighted average of codes 501 & 502, total)
Outpatient visit nurse or midwife	73·0	NHS reference cost 2017-18 (560, non-consultant led)
Outpatient physiotherapy	55·0	NHS reference cost 2017-18 (code 650)
Hospital readmissions		
Stay including intensive care	936·9	NHS reference cost 2017-18 (weighted average of codes XC03Z - XC07Z)
Non-elective short stay	640·3	NHS reference cost 2017-18 (weighted average of codes NZ26A & NZ26B)

References:

- British National Formulary 2017: Available at https://bnf.nice.org.uk/medicinal-forms/co-amoxiclav.html (Last accessed: 22 January 2019)

- Curtis, L. and Burns, A. (2017). Unit Costs of Health and Social Care. University of Kent, Personal Social Services Research Unit. Available at https://www.pssru.ac.uk/project-pages/unit-costs/unit-costs-2017/ (Last accessed: 17 January 2019)

- NHS reference cost 2017-18: Available at https://improvement.nhs.uk/resources/reference-costs/#rc1718 (Last accessed: 17 January 2019)

Table S2: Comparison of characteristics of women with complete versus missing primary outcome data

	Missing primary outcome data (n = 195)	Complete primary outcome data (n = 3225)
Maternal Age (years)		
Mean (SD)	28·2 (5·97)	30·4 (5·37)
Missing	0	0
Gestational age at randomisation (weeks)		
Median (IQR)	40 (39 to 41)	40 (39 to 41)
36+0 to 37+6	18 (9)	241 (7)
38+0 to 39+6	68 (35)	1055 (33)
40+0 to 41+6	104 (53)	1828 (57)
>=42+0	5 (3)	100 (3)
Missing	0	1
Ethnic group*		
White	164 (84)	2746 (85.5)
Indian	2 (1)	68 (2)
Pakistani	7 (4)	120 (4)
Bangladeshi	3 (2)	19 (0.6)
Black Caribbean	1 (0.5)	13 (0.4)
Black African	5 (3)	56 (2)
Any other ethnic group	13 (7)	188 (6)
Missing	0	15
Body mass index at booking (kg/m^2)		
Median (IQR)	24 (22 to 30)	25 (22 to 28)
<18·5	8 (4)	86 (3)
18·5 to 24·9	93 (49)	1600 (51)
25 to 29·9	45 (24)	861 (27)
30 to 34·9	32 (17)	391 (12)
35 to 39·9	9 (5)	142 (5)
>=40	2 (1)	64 (2)
Missing	6	81
Twin pregnancy	3 (2)	17 (0.5)
Any previous pregnancies >=22 weeks' gestation	57 (29)	718 (22)
Missing	0	4
Previous caesarean section	15 (8)	245 (8)
Missing	0	5
Previous episiotomy	20 (10)	268 (8)
Missing	1	50
Previous tear	17 (9)	144 (5)
Missing	1	49
Rupture of membranes before delivery	195 (100)	3180 (99)
<24 hours	178 (91)	2749 (85)
>=24 to <48 hours	14 (7)	352 (11)
>=48 hours	1 (0·5)	70 (2)
Unknown	2 (1)	9 (0·3)
Missing	0	0
Labour induction	101 (52)	1570 (49)
Missing	0	0
Actual mode of birth*		
Spontaneous	0	10 (0.3)
Forceps	129 (65)	2105 (65)
Ventouse	69 (35)	1127 (35)
Caesarean section	0	0
Missing	0	0
Sequential instruments used	7 (4)	148 (5)
Missing	0	0
Reason for instrumental delivery (non-exclusive)		
Failure to progress	82 (42)	1643 (51)
Fetal compromise	111 (57)	1567 (49)
Other medical reason	16 (8)	249 (8)
Missing	0	2
Episiotomy in current delivery	174 (89)	2870 (89)
Missing	0	0
Tear in current delivery	50 (26)	1003 (31)
Missing	0	0
Wound sutured	184 (99)	3126 (99)
Missing	9	78
Location suturing carried out		
Operating theatre	61 (33)	1098 (35)
Delivery ward/room	123 (67)	2027 (65)
Missing	11	100

*Includes 20 sets of twins, thus 3,440 births in total

Table S3: Sensitivity analysis: Primary outcome restricted to women who had not received antibiotics in the seven days prior to delivery

	Amoxicillin and clavulanic acid (n = 1523)	Placebo (n = 1535)	Risk ratio (95% CI)	P-value
Confirmed or suspected maternal infection	155 (11)	281 (19)	0·55 (0·46, 0·66)	<0·0001
Missing	88	93	NA	NA
Confirmed systemic infection on culture	10 (0·7)	22 (1·4)	0·46 (0·22, 0·96)	0·035
Missing	1	1	NA	NA
Endometritis	13 (0·9)	22 (1·4)	0·60 (0·30, 1·18)	0·131
Missing	0	1	NA	NA
New prescription of antibiotics with relevant indication	155 (11)	281 (19)	0·55 (0·46, 0·66)	<0·0001
Missing	88	93	NA	NA

NA Not Applicable

Table S4: Sensitivity analysis: Primary outcome restricted to women who had not been given antibiotics within 24 hours of delivery

	Amoxicillin and clavulanic acid (n = 1692)	Placebo (n = 1676)	Risk ratio (95% CI)	P-value
Confirmed or suspected maternal infection	173 (11)	300 (19)	0·57 (0·48, 0·68)	<0·0001
Missing	96	97	NA	NA
Confirmed systemic infection on culture	7 (0·4)	21 (1·3)	0·33 (0·14, 0·77)	0·0073
Missing	1	1	NA	NA
Endometritis	15 (0·9)	23 (1·4)	0·65 (0·34, 1·23)	0·181
Missing	0	1	NA	NA
New prescription of antibiotics with relevant indication	173 (11)	300 (19)	0·57 (0·48, 0·68)	<0·0001
Missing	96	97	NA	NA

NA Not Applicable

Table S5: Sensitivity analysis: Primary outcome restricted to women whose primary outcome was obtained from telephone script or questionnaire completed between 6 and 10 weeks post-delivery

	Amoxicillin and clavulanic acid (n = 1354)	Placebo (n = 1343)	Risk ratio (95% CI)	P-value
Confirmed or suspected maternal infection	145 (11)	240 (19)	0·60 (0·49, 0·72)	<0·0001
Missing	92	93	NA	NA
Confirmed systemic infection on culture	9 (0·7)	21 (1·6)	0·42 (0·20, 0·92)	0·026
Missing	0	1	NA	NA
Endometritis	12 (0·9)	19 (1·4)	0·63 (0·31, 1·29)	0·198
Missing	1	1	NA	NA
New prescription of antibiotics with relevant indication	145 (11)	240 (19)	0·60 (0·49, 0·72)	<0·0001
Missing	92	93	NA	NA

NA Not Applicable

Table S6: Sensitivity analysis: Primary outcome analysis using centre as random effect

	Amoxicillin and clavulanic acid (n = 1715)	Placebo (n = 1705)	Risk ratio (95% CI)	P-value
Confirmed or suspected maternal infection	180 (11)	306 (19)	0·58 (0·50, 0·68)	<0·0001
Missing	96	99	NA	NA
Confirmed systemic infection on culture	11 (0·6)	25 (1·5)	0·44 (0·24, 0·81)	0·0086
Missing	1	1	NA	NA
Endometritis	15 (0·9)	23 (1.3)	0·65 (0·39, 1·08)	0·094
Missing	1	1	NA	NA
New prescription of antibiotics with relevant indication	180 (11)	306 (19)	0·58 (0·50, 0·68)	<0·0001
Missing	96	99	NA	NA

NA Not Applicable

Table S7: Post hoc subgroup analysis of composite primary outcome by mode of instrumental delivery

	Amoxicillin and clavulanic acid (n = 1715)	Placebo (n = 1705)	Risk ratio (99% CI)
**Mode of instrumental delivery, n/N (%)**			
Forceps	135/1027 (13)	234/1074 (22)	0·62 (0·45, 0·86)
Vaccuum extraction	45/591 (8)	72/531 (14)	0·56 (0·39, 0·80)
Not included*	1	1	NA

* Two women with twins had both a forceps and ventouse delivery. The remaining 18 women with twin pregnancies were classified as forceps if they had at least one forceps delivery, and vacuum extraction if they had at least one vacuum extraction.
Primary outcome data missing for 126 women in forceps group and 69 women in vacuum extraction group.
NA Not Applicable

Table S8: Mean (SD) healthcare resource use and total cost (UK British pounds 2017/2018) by trial arm and mean differences between trial arms (ITT population)

	Amoxicillin and clavulanic acid (n=1296)				Placebo (n=1297)					
	**n**	**Min**	**Max**	**Mean (SD)**	**n**	**Min**	**Max**	**Mean (SD)**	**Mean difference (99% CI)**	**p-value**
**Health care resource use category**										
Health care professional number of visits										
General Practitioner	1235	0	4	0·161 (0·500)	1239	0	7	0·266 (0·689)	-0·11 (-0·17, -0·04)	<0·0001
Midwife /nurse at GP practice	1240	0	23	0·115 (0·771)	1243	0	6	0·142 (0·546)	-0·03 (-0·10, 0·04)	0·298
Midwife / nurse at home	1219	0	15	0·373 (1·079)	1226	0	10	0·551 (1·189)	-0·18 (-0·30, -0·06)	<0·0001
Health visitor/district nurse	1240	0	23	0·071 (0·731)	1253	0	11	0·081 (0·550)	-0·01 (-0·08, 0·06)	0·710
Outpatient hospital number of visits	1229	0	12	0·168 (0·828)	1231	0	17	0·310 (1·042)	-0·14 (-0·24 -0·04)	<0·0002
Length of stay hospital readmissions (in days)	1234	0	29	0·079 (0·954)	1235	0	10	0·124 (0·761)	-0·05 (-0·14, 0·04)	0·192
**Health care costs**										
Total costs at 6-weeks following delivery*	1148	2.3	19084·0	102·5 (652·4)	1144	0.0	6403·0	155·1 (497·4)	-52·6 (-115·1, 9·9)	0·030

GP: general practitioner; SD: standard deviation; CI: confidence interval
* Including cost for preventive amoxicillin/clavulanic acid and any new antibiotic prescription during follow-up
Note median and quartile values are provided in table S9

Table S9: Median and quartiles healthcare resource use and total cost (UK British pounds 2017/2018) by trial arm and mean differences between trial arms

	Amoxicillin and clavulanic acid (n=1296)				Placebo (n=1297)			
	**n**	**25th percentile**	**Median**	**75th percentile**	**n**	**25th percentile**	**Median**	**75th percentile**
**Health care resource use category**								
Health care professional number of visits								
General Practitioner	1235	0	0	0	1239	0	0	0
Midwife /nurse at GP practice	1240	0	0	0	1243	0	0	0
Midwife / nurse at home	1219	0	0	0	1226	0	0	1
Health visitor/district nurse	1240	0	0	0	1253	0	0	0
Outpatient hospital number of visits	1229	0	0	0	1231	0	0	0
Length of stay hospital readmissions (in days)	1234	0	0	0	1235	0	0	0
**Health care costs**								
Total costs at 6-weeks following delivery*	1148	2·3	2·3	44·1	1144	0·0	0·0	117·7

Supplementary appendix A1: Evaluation of the ANODE Internal Pilot

An internal pilot study of 9 months commencing in September 2015 is planned to evaluate whether procedures for recruitment and data collection work effectively and efficiently, at the request of the NIHR HTA programme. The design of the pilot study will be identical to that of the substantive trial and data collected will contribute to the final analysis. The purpose of the internal pilot is to evaluate and demonstrate feasibility particularly with regard to the following aspects:

Recruitment / Uptake by women
Loss to follow-up
Functionality and ease of the randomisation process
Intervention/placebo preparation and distribution
Collection of clinical outcomes data

It is proposed that the Trial Steering Committee (TSC) meet towards the end of the pilot phase and evaluate the performance of the internal pilot study against these key metrics, and recommend to the NIHR HTA programme either that (a) the trial proceeds immediately and seamlessly into the main phase or (b) that the pilot phase continues for a fixed period of time in order to confirm feasibility (reporting back to the TSC/HTA at some new juncture), or (c) that the trial be stopped or substantively modified in consultation with the Funder.

Timetable for pilot study

**July 2015 - September 2015**	The internal pilot study will be set up in all 8 sites with staggered start dates. During this period we will obtain R&D permissions for the 8 sites and train staff in the study procedures.
**September 2015 to June 2016**	From September 2015 to June 2016 (9 months) recruitment will take place in the 8 ANODE centres.
**Late May/early June 2016**	In 2016, towards the latter stages of the internal pilot study, we will hold a Trial Steering Committee meeting to review performance in the pilot study against our specified criteria of success. The TSC will then rapidly submit recommendations to the NIHR HTA on our behalf regarding our demonstration of feasibility. This will be, of course, contingent on gaining the necessary R&D approvals and training local personnel in trial procedures.

Benchmarks of success of the internal pilot study

Recruitment target

The recruitment target for the main trial is to recruit, on average, around 186 women per month, once a 'steady state' has been achieved; this figure takes into account differences in unit size and throughput.

Milestones for study set-up:

Plan to open eight centres with a staggered start over three months i.e. two in the first month then 3 per month over three consecutive months.

Recruitment to the ANODE internal pilot study – assumptions

Recruitment to the trial overall is planned for a total of 21 months
All centres to take four months to reach stable recruitment – 25% of target in month one, 50% in month two, 75% in month three and 100% in month four
Stable recruitment reached in all eight centres by month seven
Overall sample size is 3,424 corresponding to a required recruitment rate of 23 recruits per month per centre (based on the assumptions listed)

Proposed stop-go criteria

Proposed review of recruitment and retention by the TSC towards the end of the nine month internal pilot phase (August 2016) when 1188 recruits are predicted. Potential scenarios include:
If recruitment is 75% or more $N \geq 891$), then the target is clearly achievable – TSC recommendation to HTA would be to continue directly with the main trial;
If recruitment is 50-75% ($594 \leq N < 891$) the TSC recommendation to HTA would be to recruit more centres and review again in 6 months;
If recruitment is <50% ($N < 594$) then urgent discussions required between the Project Management Group and the TSC to undertake a detailed review of options to subsequently recommend to the HTA.

Loss to follow-up rate
The loss to follow up rate for the primary outcome is expected to be in the region of 5% for short-term outcomes up to six weeks. Therefore in a pilot study of 1188 women, we would expect <60 to be lost to follow-up; however, if no more than 64 women are lost to follow-up, this would be considered still 'reasonable' and meeting 'go-criteria' (based on the upper limit of the 95% CI to indicate the maximum number which would still be compatible with a LTFU rate of 5% {49/978 = 5% 95% CI 3.7–6.6%).

Functionality and ease of the randomisation process
Women will be randomised by the allocation of the next sequentially numbered box once consent and eligibility are established. Functionality of the randomisation process will be evaluated against the following criteria:

- Breaches of eligibility
- Timing of randomisation
- Time taken to randomise
- Use of intervention pack in correct sequential order within centre

Intervention and placebo preparation and distribution
Eligible women will be randomised to receive a single intravenous dose of prophylactic co-amoxiclav or placebo by the allocation of the next sequentially numbered box once consent and eligibility are established.

The research midwife, clinicians and the women will remain masked to allocation (note that the research midwife will be collecting outcomes information). The person responsible for administering the trial drug who may be, for example, a doctor, midwife, nurse, Operating Department Practitioner (OPD) or other healthcare professional (centre-dependant), will not be blinded to allocation (these people will not be involved in the collection of outcomes information).

The acceptability of the intervention and placebo will be evaluated through:

- Feedback from clinical staff
- Any reported deviations from stipulated dosing regimen
- Timing of the administration of the trial intervention
- Discrepancies in the IMP supply to sites
- Reconciliation of unused IMP
- Any reported breach in masking of the intervention
- Instances of unblinding

Collection of clinical outcomes and event rates
Staff at centres will complete electronic data collection forms (eDCFs) for each woman enrolled in the study. The acceptability of the data collection process will be evaluated by considering:

- Completeness: the proportion of due forms returned
- The ease of completion and acceptability of eDCFs for clinical staff
- The timing of antibiotic prescriptions (within or more than 24 hours post-delivery)
- Primary outcome rate
- Safety monitoring reports

Supplementary appendix A2: Summary of interim and final analysis of the composite primary outcome presented to the DMC

Date of DMC meeting	Date of data snapshot	Arm X	Arm Y	Risk ratio (95% CI)	p-value
7 Feb 2017	4 Jan 2017	56/198 (28%)*	55/194 (28%)*	1·00 (0·73 to 1·37)	0·988
22 Jun 2017	10 Jun 2017	41/535 (8%)	83/519 (16%)	0·48 (0·34, 0·68)	Not provided
27 Nov 2017	10 Jun 2017	63/515 (12%)	92/501 (18%)	0·67 (0·50, 0·90)	Not provided
15 Feb 2018	21 Jan 2018	90/928 (10%)	156/864 (18%)	0·54 (0·42, 0·68)	<0·001
14 Jan 2019 (final analysis)	11 Dec 2018	180/1619 (11%)	306/1606 (19%)	0·58 (0·46, 0·69)	<0·001

*Note the change in primary outcome definition occurred following the first DMC meeting.

There were no formal efficacy stopping rules applied, only a guideline of a difference of at least 3 standard errors, at which point a wider discussion about continuing would be prompted. This guideline was based broadly on the Haybittle-Peto approach i.e. proof beyond reasonable doubt, where a very stringent critical value is specified for interim analyses, but no alpha-spending function was pre-specified with threshold p-values in the DMC Charter.

Table S10: Treatment effect on primary outcome by centre

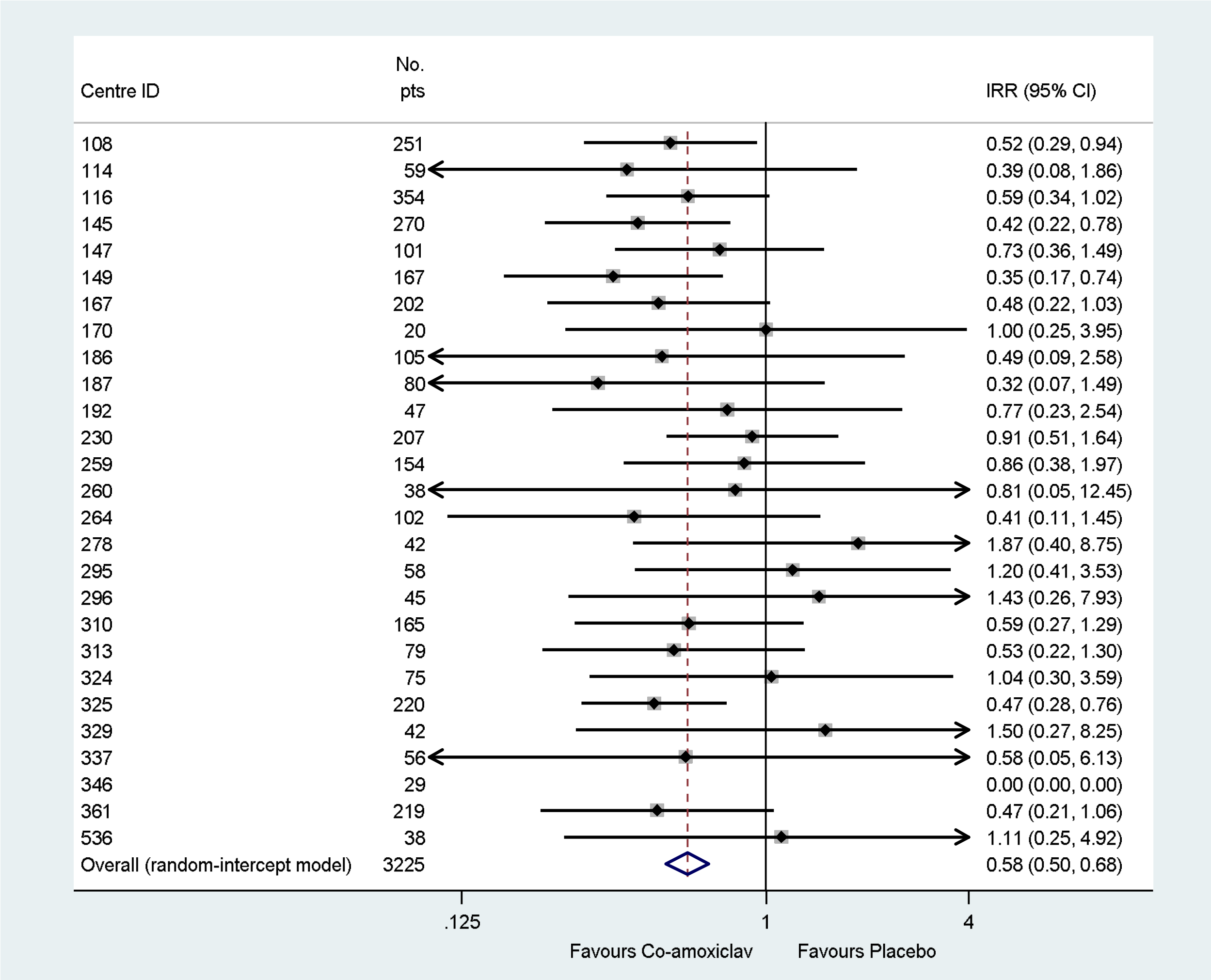

Data Monitoring Committee and Trial Steering Committee members

Data Monitoring Committee:

Professor Siladitya Bhattacharya, Chair, Head of Division of Applied Health Sciences and Director Institute of Applied Health Sciences, School of Medicine and Dentistry, University of Aberdeen

Professor Carrol Gamble, Professor of Medical Statistics, Department of Biostatistics, University of Liverpool

Dr Michael Millar, Consultant Microbiologist, Barts and the London Pathology & Pharmacy Building, London

Trial Steering Committee:

Dr Fiona Denison, Chair, Honorary Consultant in Maternal and Fetal Medicine, MRC University of Edinburgh Centre for Reproductive Health, University of Edinburgh

Mr Timothy Overton, Consultant in Obstetrics and Fetal Medicine, St Michael's Hospital, Bristol

Dr Matthew Wilson, Consultant in Anaesthesia, Royal Hallamshire Hospital, Sheffield

Ms Rachel Plachinski, NCT Research Engagement Officer, NCT Excellent Practitioner (Antenatal) and Assessor, Patient & Public Involvement (PPI) representative

Ms Donna Southam, Audit & Research Midwife, Maternity Unit Basildon Hospital, Essex

Professor Simon Cousens, Professor of Epidemiology and Medical Statistics and Research Degrees Director, London School of Hygiene & Tropical Medicine, London
